# Distinct Microbiota and Functional Pathway Profiles Define Success and Failure in Regenerative Endodontic Treatment

**DOI:** 10.1111/iej.70205

**Published:** 2026-06-23

**Authors:** Leimarembi Devi Naorem, Alina Wikström, Peter Quach, Lokeshwaran Manoharan, Ronald Ordinola‐Zapata, Olena Rakhimova, Malin Brundin, Nelly Romani Vestman

**Affiliations:** ^1^ Department of Odontology Umeå University Umeå Sweden; ^2^ Department of Endodontics, Public Dental Health Services Eastman Institute Stockholm Sweden; ^3^ Division of Paediatric Dentistry, Department of Dental Medicine Karolinska Institute Stockholm Sweden; ^4^ Centre of Paediatric Oral Health Huddinge Sweden; ^5^ Region of Västerbotten Umeå Sweden; ^6^ Region of Uppsala Uppsala Sweden; ^7^ National Bioinformatics Infrastructure Sweden (NBIS) Lund University Lund Sweden; ^8^ Division of Endodontics, Department of Restorative Sciences, School of Dentistry University of Minnesota Minneapolis Minnesota USA; ^9^ Department of Medical Biosciences Umeå University Umeå Sweden

**Keywords:** 16S rRNA gene sequencing, calcium hydroxide, differential abundance, microbiota, regenerative endodontic treatment, treatment outcome

## Abstract

**Aim:**

To characterize the intracanal microbiota and identify key microbial taxa and functional pathways associated with regenerative endodontic treatment (RET) outcomes.

**Methodology:**

In this observational cohort study, 196 samples were collected at five time points from external tooth surfaces (T1, T4) and root canals (T2, T3, T5). The V3–V4 hypervariable region of 16S rRNA gene was amplified and sequenced on the Illumina MiSeq platform. Sequence data were pre‐processed using QIIME2 and DADA2 to generate amplicon sequence variants (ASVs), classified against the eHOMD database. Microbial diversity, differential abundance, and Receiver Operating Characteristic (ROC) analyses were performed using R statistical software. Functional profiles were predicted using PICRUSt2.

**Results:**

RET induced dynamic shifts in the root canal microbiome, with initially predominant phyla including Actinomycetota, Bacillota, Bacteroidota, Fusobacteriota and Pseudomonadota. Cases with treatment failure consistently showed a higher relative abundance of Fusobacteriota than successful cases. Alpha diversity varied across time points, with outcome‐specific differences observed only at T3; beta diversity differed significantly by time point but not by treatment outcome. Differential abundance analysis at T5 revealed enrichment of *Rothia dentocariosa* and *Leptothrix* sp. in successful cases, whereas failed cases were enriched in *Fusobacterium polymorphum*, *Streptococcus sanguinis* , *Campylobacter showae* , *Lachnoanaerobaculum* sp. and *Selenomonas* sp. These taxa demonstrated moderate diagnostic potential, with area under the curve (AUC) values of 0.70–0.82. Persistence analysis showed that *F. polymorphum*, *C. showae* and *Selenomonas* sp. exhibited greater persistence in failed cases across treatment time points. Predicted functional profiling indicated distinct metabolic potentials between successful and failed treatment groups.

**Conclusions:**

RET alters the intracanal microbiome, with taxa identified following intracanal dressing exhibiting moderate predictive potential as biomarkers for treatment outcome, thereby contributing to early prognosis and informing RET strategies.

## 1 Introduction

The oral microbiome is one of the most diverse microbial ecosystems in the human body, including bacteria, archaea, fungi and viruses that help maintain oral homeostasis and contribute to host immunity and metabolism (Kilian et al. 2016; Deo and Deshmukh 2019). Disruption of this balance may lead to the onset of oral diseases such as caries, periodontitis and endodontic infections (Mosaddad et al. 2019; Wieczorkiewicz et al. 2025). When the dental pulp is exposed due to trauma, caries, or restorative procedures, the root canal can serve as a niche for microbial growth, which may lead to pulp necrosis and apical periodontitis (A. F. Fouad 2019). These primary endodontic infections are polymicrobial biofilm‐driven diseases, typically dominated by anaerobic taxa as oxygen tension decreases in the necrotic pulp milieu (Narayanan and Vaishnavi 2010; Sharaf et al. 2022).

The endodontic management of immature permanent teeth with pulp necrosis presents unique challenges. Thin dentinal walls, open apices, and wide dentinal tubules allow bacteria to penetrate deeper, making effective disinfection and obturation difficult and predisposing to root fracture and treatment failure (Kakoli et al. 2009; A. F. Fouad 2011). Although calcium hydroxide (CaOH_2_) or mineral trioxide aggregate (MTA)—based apexification remains a conventional option (Sheehy and Roberts 1997; Witherspoon et al. 2008), this treatment does not promote continued root maturation, leaving these teeth structurally compromised (Mohammadi and Dummer 2011).

Regenerative endodontic treatment (RET) has evolved as a biologically based approach that enables continued root maturation by harnessing the regenerative potential of apical papilla stem cells (Huang et al. 2008; Kim et al. 2018). Nevertheless, RET outcomes remain highly variable, with success rates ranging from 50% to 98%, depending on infection severity and disinfection effectiveness (Rojas‐Gutiérrez et al. 2022; Almutairi et al. 2019). Persistent intracanal infection is considered a significant contributing factor to failures, particularly in traumatized immature teeth (Wikström et al. 2022).

Our recent randomized clinical trial on traumatized immature incisors revealed that a substantial reduction in intracanal bacterial load was strongly associated with successful RET outcomes, regardless of whether calcium hydroxide or chlorhexidine was used as the intracanal medicament (Wikström et al. 2025). However, in some cases, RET failed despite significant bacterial reduction, and the presence of *Enterococcus faecalis* was strongly associated with RET failure, indicating that microbial composition and its functional behaviour—rather than total bacterial load alone—could play a critical role in healing. Such studies emphasize the requirements of advanced microbial profiling to characterize microbial community dynamics and metabolic pathways, specifying taxa and prognostic markers influencing RET outcomes.

Microbiome sequencing analysis, including 16S rRNA next‐generation sequencing (NGS), enables detailed analysis of microbial communities in endodontic infections, surpassing the sensitivity of culture‐based methods (Li et al. 2010; Siqueira et al. 2012; Wensel et al. 2022). These technologies have revealed highly complex microbial ecosystems in necrotic root canals, including low‐abundance or previously undetected taxa. Hence, understanding how microbial composition and functional characteristics differ between successful and failed treatment cases can reveal predictive biomarkers that enable earlier identification of high‐risk cases and improve treatment protocols (Sellami et al. 2023; Dioguardi et al. 2020).

Therefore, this study aims to comprehensively characterize the root canal microbiome in immature necrotic traumatized incisors treated with calcium hydroxide‐based RET using 16S rRNA sequencing. Based on microbial diversity, taxonomic signatures, and predicted functional pathways, we aim to uncover resilient pathogenic taxa, beneficial microbial markers, and metabolic activities that contribute to treatment outcomes. Such microbial biomarkers could serve as early predictors of prognosis and guide tailored therapeutic strategies to improve clinical outcomes in RET.

## 2 Materials and Methods

### 2.1 Ethics Statement

The study was reviewed and approved by the Stockholm Central Ethical Review Board (Dnr: 2018/692‐31), the Swedish Ethical Review Authority (Ref: 2022‐05715‐02) and the Regional Ethical Board at Umeå University, Sweden (Dnr: 2016/520‐31). Before treatment, participants and their legal guardians received both oral and written information about the study, and written informed consent was obtained. The study was conducted in accordance with the Declaration of Helsinki and Swedish regulations. The study is reported under the Strengthening the Reporting of Observational studies in Epidemiology (STROBE) guidelines.

### 2.2 Study Population and Sample Collection

Participants aged 7–18 with traumatized permanent incisors with immature root development and a diagnosis of pulp necrosis with or without apical periodontitis were recruited at the specialist clinics in endodontics and paediatric dentistry at the Eastman Institute, Stockholm, and the Department of Endodontics, Umeå University, Sweden. Detailed inclusion and exclusion criteria have been described previously (Wikström et al. 2022). All 61 enrolled patients underwent RET using calcium hydroxide as an intracanal medicament. A total of 196 microbial samples were collected across five treatment time points and stratified by treatment outcomes (success and failure): T1‐tooth surface sample (contamination control) at the first visit (*n* = 53; success = 41, failure = 12), T2‐root canal sample before debridement (*n* = 56; success = 44, failure = 12), T3‐root canal sample after debridement (*n* = 29; success = 22, failure = 7), T4‐tooth surface sample (contamination control) at the second visit (*n* = 29; success = 22, failure = 7) and T5‐root canal sample after root canal dressing (calcium hydroxide) (*n* = 29; success = 22, failure = 7) using procedures previously described (Wikström et al. 2024). The final sample numbers at each time point reflect the exclusion of samples that failed quality control due to insufficient or degraded DNA. Treatment outcomes (success or failure) were evaluated according to clinical and radiographic criteria 48–57 months following treatment completion (Wikström et al. 2022).

### 2.3 Tooth Surface Disinfection and Sampling

Following local anaesthesia, the tooth was isolated with a rubber dam and retainer. Access was initiated through enamel using a high‐speed bur. The crown, retainer and rubber dam were disinfected with sterile foam pellets soaked in 30% H_2_O_2_ and 5% chlorhexidine–ethanol and subsequently neutralized with 5% sodium thiosulfate. Decontamination efficacy was verified by sampling the disinfected crown with two foam pellets (T1). One pellet was placed in Tris‐EDTA buffer (10 mmol/L Tris hydrochloride, 1 mmol/L EDTA) for DNA analysis, and the other in 5 mL fluid thioglycolate medium supplemented with agar. All samples were transferred to Tris‐EDTA buffer to stabilize nucleic acids for molecular analyses.

### 2.4 Clinical and Microbiological Procedures

**FIGURE 1:**
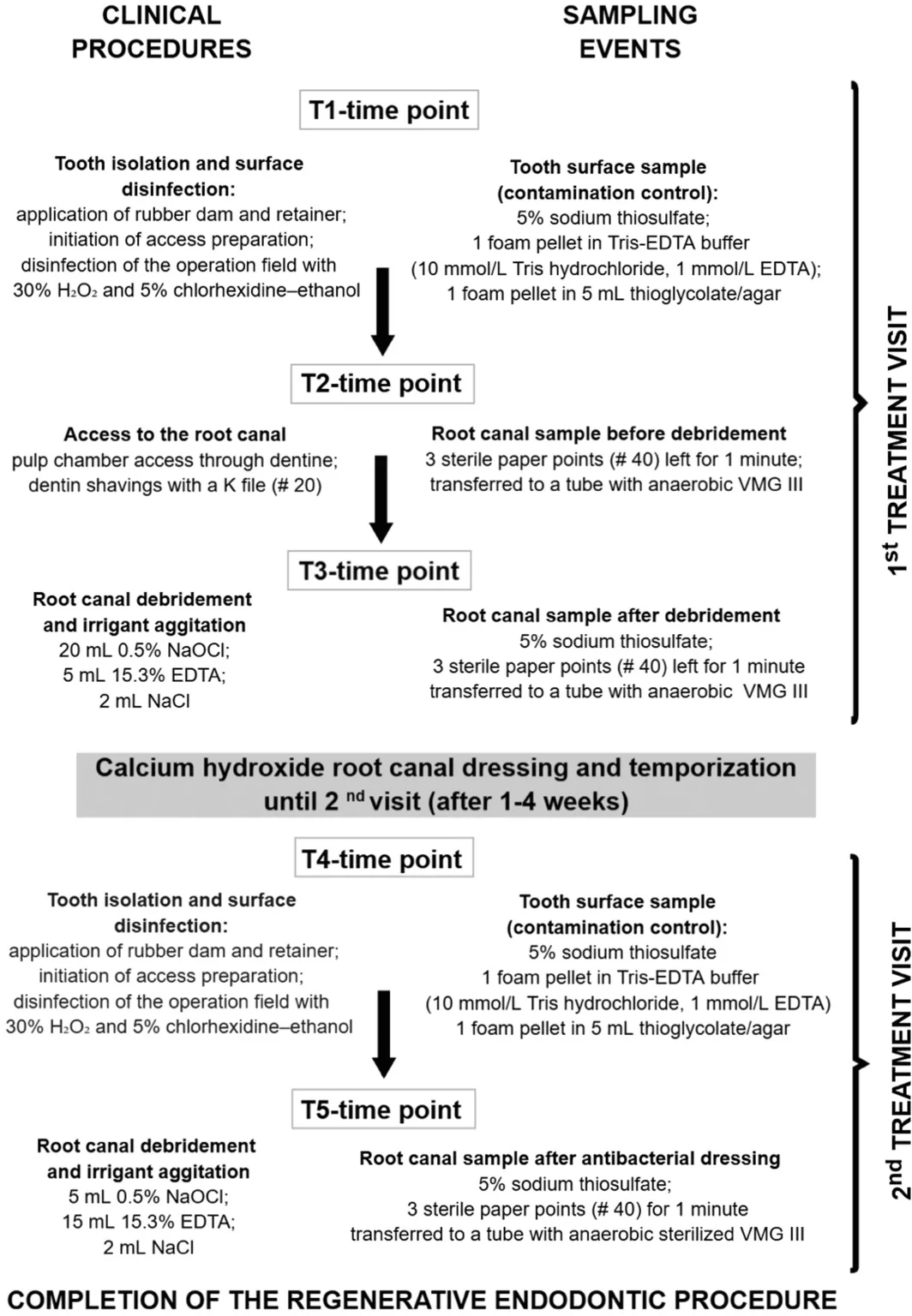
Description of the treatment time points (T1–T5).

The pulp chamber was accessed through dentine with a sterile bur. A root canal sample (T2) was obtained prior to instrumentation or irrigation. Sterile saline was introduced to the level of the cementoenamel junction. Dentine shavings were collected using a sterile K‐file (size 20) to disrupt adherent biofilm. Canal contents were absorbed with three sterile paper points (ISO 40) placed to working length for 1 min and transferred to reduced transport medium (anaerobically sterilized VMG III). In accordance with European Society of Endodontology recommendations (Galler et al. 2016), mechanical instrumentation was avoided due to the fragile structure of immature teeth. Disinfection was achieved by ultrasonic agitation of irrigants using IrriSafe files (Acteon). The root canal was irrigated with 20 mL of 0.5% buffered sodium hypochlorite (Dakin's solution, APL, Apoteket AB, Stockholm, Sweden) and with 5‐mL of ethylenediaminetetraacetic acid (EDTA, 15.3% APL, Apoteket AB, Stockholm, Sweden). After completing debridement, the root canal irrigants were inactivated with 1 mL sodium thiosulfate for 1 min, which was retained in the canal during sampling. Dentine shavings were obtained using a sterile K‐file (size 20) to disrupt adherent biofilm. A root canal sample (T3) was collected as described above and transferred to VMG III. An intracanal medicament was placed using a sterile syringe and distributed with a Lentulo spiral. The access cavity was temporized with ≥ 4 mm of temporary restoration consisting of zinc oxide–eugenol cement and glass ionomer. A second visit was scheduled after approximately 4 weeks. At the second visit, if no clinical signs or symptoms of persistent infection were present, the revitalization procedure was initiated. The temporary restoration was removed, the tooth was isolated with a rubber dam, and disinfected as previously described. A contamination control sample (T4) was collected following the same procedure as T1. The intracanal medicament was inactivated with 1 mL sodium thiosulfate for 1 min, followed by irrigation with 2 mL sterile saline. Dentine shavings were then obtained using a sterile K‐file (size 20), and a root canal sample (T5) was collected as described for S3. The canal was irrigated with 5 mL 0.5% buffered sodium hypochlorite, followed by 15 mL 15.3% EDTA for 5 min, and a final rinse with 2 mL sterile saline. The canal was dried with sterile paper points. Bleeding was induced from the periapical tissues using a pre‐curved file (K‐file size 15 or Hedström file size 30) until the canal was filled to the cemento‐enamel junction. After approximately 15 min to allow clot stabilization, a resorbable collagen scaffold was placed, followed by ≥ 2 mm of bioceramic material at the cemento‐enamel junction. Materials included mineral trioxide aggregate (white MTA Pro Root, Dentsply, Maillefer, Switzerland), biodentine (Septodont), or bioceramic root repair condensable putty (EndoSequence BC RRM; Brasseler). A glass ionomer layer was placed, and the tooth was definitively restored with resin composite at the same visit. The five treatment time points are summarized in Figure 1.

### 2.5 Outcome Assessment

Treatment outcomes (success or failure) were evaluated according to clinical and radiographic criteria at 48–57 months following treatment completion (Wikström et al. 2022). Success was defined as the absence of clinical signs and symptoms (pain, sinus tract, swelling, or sensitivity to palpation or percussion), accompanied by radiographic evidence of apical healing and continued root development. Failure was defined as the presence of clinical symptoms after completion of the procedure or radiographic evidence of persistent apical periodontitis, lack of continued root formation (length or width), incomplete apex closure, or persisting/progressive resorption, as assessed at the final follow‐up. Post‐operative failures were managed by apexification with apical plug technique using bioceramic materials.

### 2.6 Genomic DNA Isolation

Genomic DNA was extracted from bacterial samples using the GenElute Bacterial Genomic DNA Kit (Sigma Aldrich, NA2120) following the manufacturer's protocol, with an upstream enzymatic lysis step. Briefly, cell pellets were pre‐treated with a lysozyme (Merck, L6876‐5G) and mutanolysin (Merck, M9901‐10KU) solution to ensure efficient bacterial cell lysis. Purified DNA concentration was measured using a NanoDrop instrument (Thermo Fisher Scientific; 01‐058112), and samples were stored at −20°C before 16S rRNA gene amplicon sequencing.

### 2.7 Amplicon Generation and Library Preparation for Sequencing

Illumina MiSeq sequencing for the study samples was performed at the HOMINGS Forsyth Institute (Cambridge, MA, USA) using the Quick‐16S NGS Library Prep Kit with the V3–V4 primer set. The V3–V4 hypervariable regions of the 16S rRNA were PCR amplified using primers 341F and 806R (Johansson et al. 2018). Real‐time PCR reactions were monitored to minimize chimera formation, and final products were quantified by qPCR, pooled equimolarly, cleaned with Select‐a‐Size DNA Clean & Concentrator, and quality‐checked with TapeStation and Qubit. The pooled library was sequenced on an Illumina MiSeq platform using a v3 reagent kit with a 10% PhiX spike‐in.

### 2.8 Data Processing and Taxonomy Profiling

Raw, demultiplexed paired‐end FASTQ reads were processed and analysed using Quantitative Insights Into Microbial Ecology version 2 (QIIME2) (Bolyen et al. 2019). V3–V4 16S rRNA primers (Forward: CCTACGGGAGGCAGCAG; Reverse: GGACTACHVGGGTWTCTAAT) were first removed from the reads using cutadapt plugin (Martin 2011), with a maximum error rate of 0.15. Following primer trimming, reads were processed using Divisive Amplicon Denoising Algorithm 2 (DADA2) plugin in QIIME2 (Callahan et al. 2016). This step included quality control, denoising, paired‐end read merging, and chimera removal, resulting in a feature table of Amplicon Sequence Variants (ASVs).

Due to technical and time constraints, the samples were processed across two independent sequencing runs. Within each run, reads were trimmed based on quality scores, filtered according to expected error thresholds, and analysed using a pseudo‐pooling strategy to improve detection of rare variants. ASV tables from both runs were subsequently merged before downstream analysis. The final dataset comprised samples with sequencing depths ranging from 10 564 to 197 359 reads. To ensure data quality and minimize noise, low‐prevalence features were filtered by retaining ASVs present in at least two samples with a minimum frequency of 10 reads. Taxonomic classification was assigned to representative ASV sequences using a Naive Bayes classifier trained on the expanded Human Oral Microbiome Database (eHOMD, version 16.01) (Chen et al. 2010).

### 2.9 Alpha and Beta Diversity Analysis

To account for differences in sequencing depth, rarefaction was applied for beta‐diversity analyses using the minimum sample sequencing depth. Alpha diversity (intra‐sample diversity) was assessed using four indices: Observed species and Chao1 (species richness), while Shannon and Inverse Simpson (InvSimpson) indices (species richness and evenness). These metrics were calculated using *phyloseq* R package (v1.52.0) (McMurdie and Holmes 2013). Group comparisons were performed using Wilcoxon rank‐sum test for binary variables and Kruskal–Wallis test for multi‐group comparisons (Kruskal and Wallis 1952). Where Kruskal–Wallis test indicated significance, pairwise comparisons were performed using Dunn's post hoc test with *p*‐values adjusted via Bonferroni method. Beta diversity (between‐sample) was evaluated using Bray–Curtis dissimilarity with *vegan* R package (v2.6.10) (Dixon 2003) and visualized using Principal coordinate analysis (PCoA) (Halko et al. 2011). Permutational multivariate analysis of variance (PERMANOVA) was performed separately for each time point using adonis2 function with 9999 permutations to assess the effects of treatment outcomes and clinically relevant covariates (Anderson 2017). To assess potential batch effects, sequencing run was included as a factor in PERMANOVA. Although statistically significant, batch explained only a small proportion of the variance (*R* ^2^ = 0.01). Because the distribution of samples across sequencing runs was highly unbalanced, batch correction was not applied to avoid introducing potential bias.

### 2.10 Differentially Abundant Microbes

Differentially abundant microbial species between successful and failed treatment outcomes were identified using Analysis of Compositions of Microbiomes with Bias Correction 2 (ANCOM‐BC2) (Lin and Peddada 2020), Microbiome Multivariable Association with Linear Models (MaAsLin2) (Mallick et al. 2021) and Linear Discriminant Analysis Effect Size (LEfSe) (Segata et al. 2011). ANCOM‐BC2 analysis (ANCOM‐BC2 R package) accounted for compositionality and sequencing bias, with treatment outcome as the primary variable. Parameters included false discovery rate (FDR) adjustment, prv_cut = 0.1 and structural zero detection. MaAsLin2 analysis (MaAsLin2 R package) employed multivariable linear models (LM) on Total Sum Scaling (TSS)—normalized and log‐transformed counts, adjusting for significant covariates. Parameters included correction = FDR, analysis_method = LM, min_abundance = 0.001 and min_prevalence = 0.1. LEfSe analysis (microbiomeMarker R package) retained features present in at least 10% of samples, followed by normalization to Counts Per Million (CPM). Statistical significance was assessed using Wilcoxon rank‐sum test (*p* < 0.05), followed by Linear Discriminant Analysis (LDA) to estimate effect sizes. Features with LDA scores (log_10_) > 3.0 were considered potential biomarkers.

### 2.11 Microbiota Persistence Across Treatment Outcomes

Microbial persistence was assessed using paired samples from 25 patients (18 successes and 7 failures). Persistence was evaluated at two time point pairings (T1 → T5 and T2 → T5) and defined as the presence of a microbial species in both samples from the same patient. Fisher's exact test was used to compare persistence frequencies between treatment groups. Odds ratios (OR) with 95% confidence intervals were calculated to assess the strength of association. Species with *p* < 0.05 were considered statistically significant.

### 2.12 Receiver Operating Characteristic Evaluation of Microbial Species Associated With Treatment Outcomes

To evaluate the predictive potential of significant microbial species, Receiver Operating Characteristic (ROC) curves were generated using the pROC R package (Robin et al. 2011). The Area Under the Curve (AUC), along with 95% confidence intervals, was computed using ci.auc() for T2 and T5 to assess discriminative performance. Optimal cutoffs were determined using Youden's index (sensitivity + specificity − 1) to estimate diagnostic sensitivity and specificity.

### 2.13 Functional Prediction and Analysis

The functional potential of the microbiome was predicted from 16S rRNA data using Phylogenetic Investigation of Communities by Reconstruction of Unobserved States 2 (PICRUSt2 v2.5.2) tool (Douglas et al. 2020). Predicted functions were mapped to metabolic pathways using the MetaCyc database. Differential abundance analysis of these pathways was performed using the edgeR method implemented in the ggpicrust2 R package (v1.7.3) (Yang et al. 2023). Pathways with adjusted *p*‐value < 0.05 were considered statistically significant.

### 2.14 Correlation Analysis

Spearman's rank correlation was used to assess relationships between microbial species and predicted functional pathways. Correlation coefficients were visualized as heatmaps, with cell colour indicating the degree and direction of the correlation. Statistically significant correlations (*p* < 0.05) were indicated with asterisks.

## 3 Results

### 3.1 Patients' Demographic and Clinical Characteristics and Data Quality Assessment

The demographic and clinical characteristics of 61 patients, stratified into success (*n* = 48) and failure (*n* = 13) treatment outcomes, are summarized in Data S1. Among the covariates assessed, trauma involving both soft and hard tissues was significantly associated with outcomes (*p* = 0.02), whereas no other demographic or clinical variables were significantly associated with outcomes. Sequencing of the 196 high‐quality samples yielded a total of 6 969 733 high‐quality non‐chimeric reads (Data S2), resulting in the identification of 1762 amplicon sequence variants (ASVs) (Data S3). Taxonomic classification of these ASVs revealed 2 kingdoms, 12 phyla, 23 classes, 45 orders, 85 families, 165 genera and 389 species. Notably, 165 ASVs (9.4%) could only be classified at the kingdom level (Bacteria) and were not assigned to lower taxonomic rank.

### 3.2 Shifts in Microbiome Composition Distinguish Treatment Success and Failure

**FIGURE 2:**
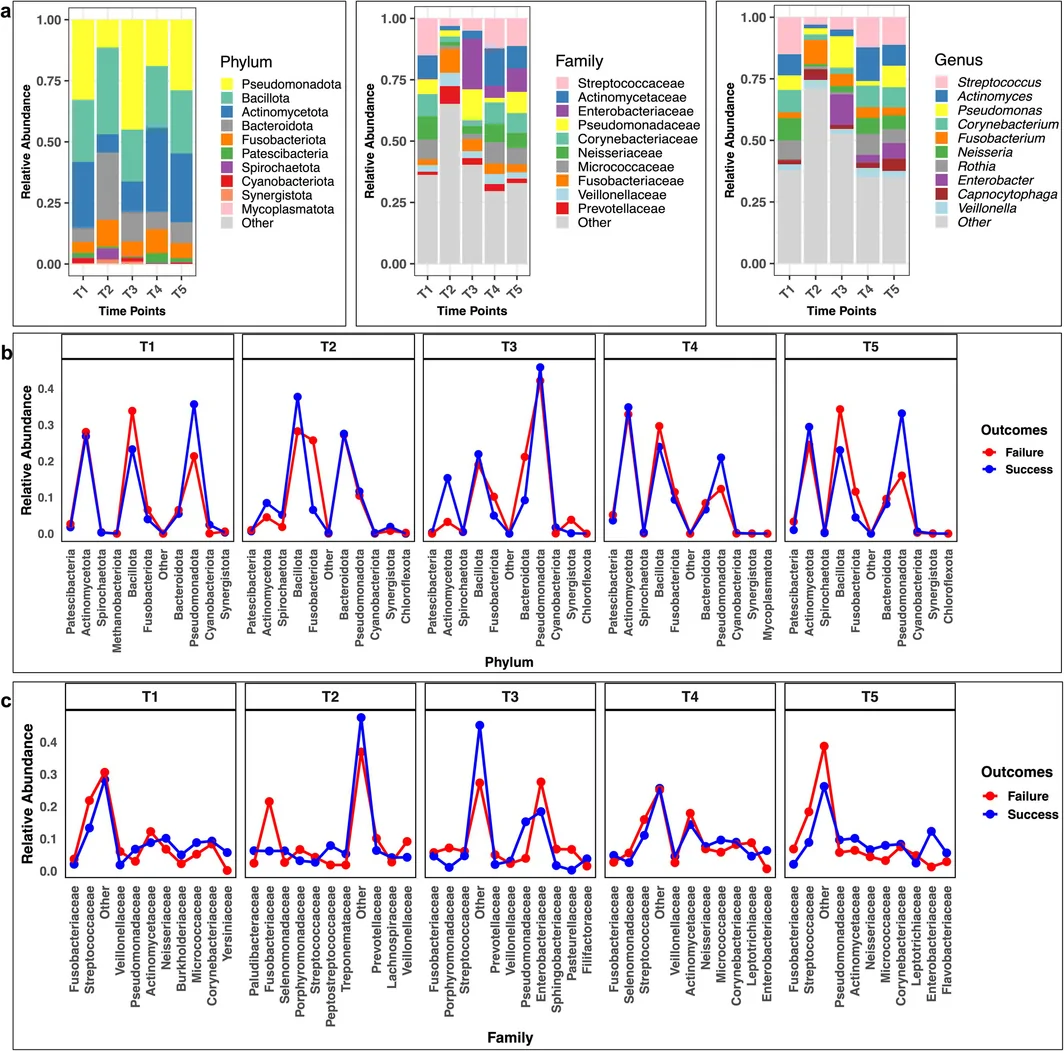
Taxonomic composition of microbial communities at all time points and outcome‐stratified comparisons. T1–T5 indicate time points including tooth surface controls (T1, T4) and root canal samples collected before debridement (T2), after debridement (T3) and after calcium hydroxide dressing (T5). (a) Stacked bar plots show the mean relative abundance of the top most abundant taxa at the phylum, family and genus levels across all samples at each time point, independent of outcome; remaining taxa are grouped as ‘Other’. (b) Line plots depict the mean relative abundance of the top phyla and (c) families at T1–T5, stratified by treatment outcomes, with taxa outside the top‐ranked groups aggregated as ‘Other’.

To analyze microbiome composition during RET, the relative abundance of bacterial taxa was evaluated across three intracanal time points (T2, T3 and T5) and two external surface controls (T1 and T4). At T2, the microbial community was predominantly composed of Bacillota, Bacteroidota, Pseudomonadota and Fusobacteriota. Fusobacteriaceae and Prevotellaceae were the dominant families, and *Fusobacterium* and *Capnocytophaga* were the dominant genera. A high proportion of taxa were present at low abundance, reflecting a highly diverse microbial community. At T3, there was a shift in the intracanal microbiota, with Pseudomonadota becoming dominant, accompanied by a reduction in Bacillota and Bacteroidota. Enterobacteriaceae and Pseudomonadaceae became predominant, while *Pseudomonas* and *Enterobacter* emerged as dominant genera, suppressing taxa that were prominent in the initial infection. At T5, the microbial community showed increased abundance of Actinomycetota. Streptococcaceae, Enterobacteriaceae and Actinomycetaceae were the most abundant families, while *Streptococcus*, *Pseudomonas*, *Actinomyces* and *Corynebacterium* dominated at the genus level. The external surface microbiota (T1 and T4) showed a consistent composition, dominated by Actinomycetota and Bacillota. Streptococcaceae and Actinomycetaceae were the most abundant families, *Actinomyces* and *Streptococcus* as leading genera (Figure 2a). Mean relative abundances (%) of microbial taxa at the phylum, family and genus levels across all time points are provided in Table S1. Sample‐wise stacked bar plots of phylum and family level relative abundances further revealed substantial inter‐individual variability at each time point (Figure S1a,b).

When analysed separately, both success and failure groups exhibited shifts in microbiome composition, with variations in relative abundance. At T2, subtle differences were observed at the phylum level, with Bacillota, Bacteroidota, Actinomycetota and Pseudomonadota more abundant in success and Fusobacteriota in the failure group. At family level, the failure group was dominated by Fusobacteriaceae, Prevotellaceae and Veillonellaceae, whereas success group contained these taxa at lower levels and exhibited greater diversity of other low‐abundance families. At T3, both groups showed a shift in community composition, with reduced Bacteroidota and Bacillota and increased dominance of Pseudomonadota. Enterobacteriaceae was particularly abundant in failure group. At T5, the success group showed higher relative abundances of Pseudomonadota and Actinomycetota with lower Fusobacteriota. Bacillota dominated the failure group, with Fusobacteriota remaining relatively abundant. At the family level, successes had higher abundances of Enterobacteriaceae, Actinomycetaceae and Pseudomonadaceae, while failures were enriched in Streptococcaceae and Fusobacteriaceae. At T1, minimal differences were observed between groups, with both dominated by Actinomycetota, Bacillota and Pseudomonadota. At the family level, Streptococcaceae and Actinomycetaceae were more abundant in failure samples. At T4, both groups were dominated by Actinomycetota and Bacillota, with higher Fusobacteriota in the failure group and higher Pseudomonadota in success group. At family level, Streptococcaceae and Actinomycetaceae were more prominent in failure group (Figure 2b,c). Sample‐wise stacked bar plots at phylum‐level further illustrate inter‐individual variability at all time points (Figure S1c).

### 3.3 Microbial Diversity Reveals Temporal Shifts but No Major Compositional Divergence Between Successful and Failed RET Cases

**FIGURE 3:**
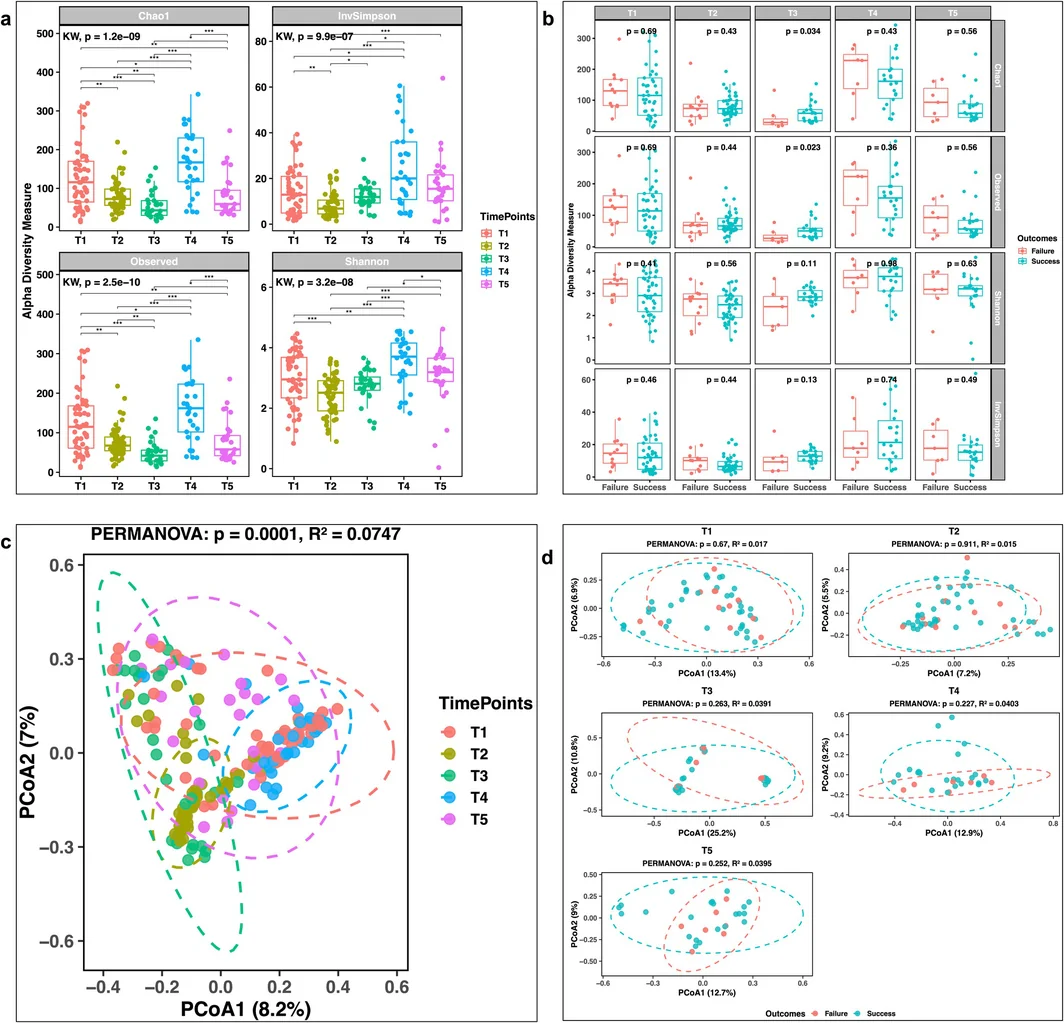
Microbial diversity and community composition across time points and treatment outcomes. T1–T5 indicate time points, including tooth surface controls (T1, T4) and root canal samples collected before debridement (T2), after debridement (T3) and after calcium hydroxide dressing (T5). (a) Boxplots of alpha diversity metrics (Chao1, Observed, Shannon and Inverse Simpson) show significant differences across time points as assessed by Kruskal–Wallis test (*p* < 0.05). Asterisks indicate significant pairwise comparisons identified using Dunn's post‐hoc test with Benjamini–Hochberg (BH) correction (*p ≤ 0.05, **p ≤ 0.01, ***p ≤ 0.001). (b) When stratified by treatment outcome, alpha diversity did not differ significantly between success and failure groups at most time points; however, at T3, Chao1 and Observed richness metrics showed significant differences (*p* < 0.05). (c) Principal coordinate analysis (PCoA) illustrated differences in microbial community composition across time points. (d) Outcome‐stratified PCoA plots indicate no significant differences in beta diversity between success and failure groups at any time point.

Alpha diversity differed significantly across time points for all four indices. Overall comparisons using Kruskal–Wallis test showed highly significant differences in Observed species, Chao1, Shannon and Inverse Simpson indices (*p* < 0.001 for all) (Figure 3a). Pairwise comparisons using Dunn's test with Benjamini‐Hochberg (FDR) correction revealed a profound and consistent difference between external and intracanal environments (Table S2). Both external tooth surface communities (T1 and T4) were significantly more diverse than intra‐canal communities (T2, T3 and T5) for both Observed and Chao1 metrics (all adjusted *p* < 0.05). Species richness analysis showed that T2 harboured a moderate number of species. Following T3, there was a statistically significant decrease in species richness compared to T2 for both Chao1 (*p* = 0.0075) and Observed (*p* = 0.0037), indicating the elimination of many bacterial taxa. Subsequently, T5 showed a slight increase in richness compared to T3 (Chao1: *p* = 0.029 and Observed: *p* = 0.011); although richness at T5 was not significantly different from T2. Shannon diversity did not change significantly between T2 and T3, whereas InvSimpson diversity increased significantly (*p* = 0.010), suggesting increased evenness among surviving taxa after debridement. From T2 to T5, both Shannon (*p* = 0.0003) and InvSimpson (*p* = 0.0002) indices increased significantly, indicating a more even community structure at T5 despite comparable richness. In contrast, T1 and T4 showed the highest levels of microbial diversity across all indices when compared to intracanal samples. T4, in particular, exhibited significantly higher richness than at T1, for Chao1 (*p* = 0.026), Observed species (*p* = 0.040) and Shannon diversity (*p* = 0.007). This resulted in a profound and statistically significant difference between the low‐diversity intracanal environment and the high‐diversity external tooth‐surface microbiome (Figure 3a).

To assess associations between microbial diversity and treatment outcomes, each time point (T1, T2, T3, T4 and T5) was stratified into success and failure groups. No statistically significant differences in alpha diversity were observed between groups at most time points, including T1, T2, T4 and T5. At T3, however, the success group showed significantly higher Chao1 richness (*p* = 0.034) and Observed species (*p* = 0.023) than the failure group. A non‐significant trend was observed at T5, where median values of Chao1, Observed and InvSimpson were higher in the failure group (Figure 3b). The association of clinical and demographic covariates and alpha diversity was assessed at each time point. At T2, trauma involving both soft and hard tissues was significantly associated with diversity. No covariates were significantly associated with alpha diversity at T5 (Data S4).

The distribution of microbial communities was visualized using PCoA, and PERMANOVA revealed a significant effect of time point on community composition (Figure 3c, PERMANOVA test, *p* = 0.0001). To assess potential confounding factors, PERMANOVA analyses were performed at each time point, including demographic and clinical covariates. Most variables were not significantly associated with microbial beta‐diversity at T2 or T5 (Table S3). When analysed by treatment outcome alone, no significant differences in beta diversity were observed at any individual time point Figure 3d. After adjusting for covariates, results remained non‐significant, suggesting that the lack of clustering by outcome may reflect the limited sample size.

### 3.4 Distinct Microbial Signatures Linked to RET Outcomes Across Time Points

The microbial taxa associated with RET outcomes were identified through differential abundance analyses at the species level across time points (T2 and T5). MaAsLin2 and ANCOM‐BC2 were used for T2 samples to account for significant covariates. For T5 samples lacking significant confounders, MaAsLin2, ANCOM‐BC2 and LEfSe were used to identify biomarkers, with LEfSe providing effect‐size rankings via LDA scores. At T2, trauma type was identified as a significant covariate influencing the microbial community. After adjusting for trauma type, ANCOM‐BC2 identified four species significantly associated with treatment outcomes (*p* < 0.05, *q* < 0.1). *Slackia exigua* and *Tannerella forsythia* were enriched in successful outcomes, whereas *Porphyromonas endodontalis* and *Fusobacterium vincentii* were enriched in treatment failures. MaAsLin2 also identified taxa with *p*‐value significance, although none remained significant after FDR correction. At T5, ANCOM‐BC2 identified *Actinomyces naeslundii* and *Lachnoanaerobaculum* sp. as enriched in failures (*p* < 0.05, *q* < 0.1). LEfSe analysis further identified *Rothia dentocariosa* and *Leptothrix* sp. in successes and *Arachnia propionica* , *Fusobacterium polymorphum*, *Campylobacter showae* and *Lachnoanaerobaculum saburreum* in failures (*p* < 0.05, LDA > 3). MaAsLin2 identified overlapping taxa with *p*‐value significance, although none remained significant after FDR correction. All taxa with *p* < 0.05 across time points and statistical methods are listed in Table S4.

### 3.5 Key Microbes as Candidate Biomarkers of Treatment Outcomes

**FIGURE 4:**
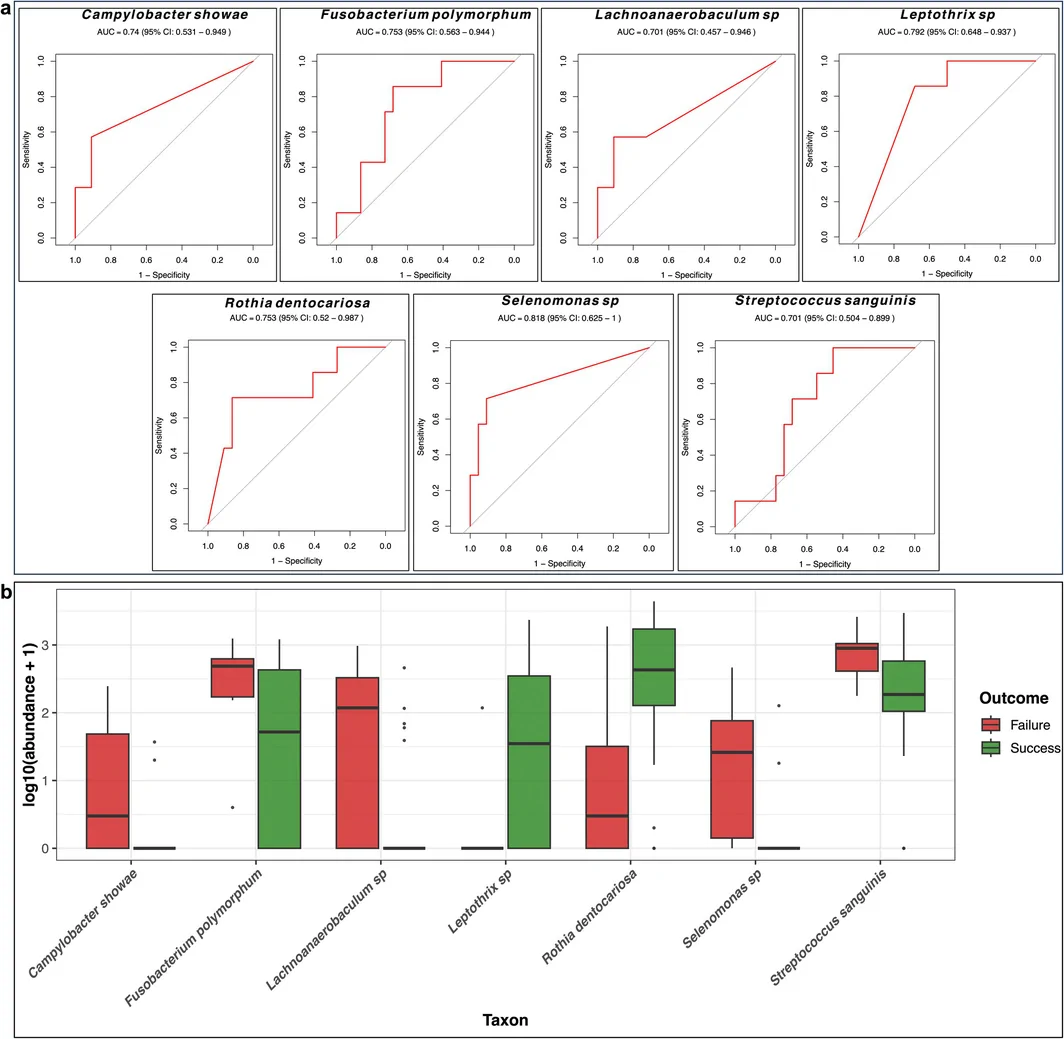
Predictive potential and differential abundance of selected microbes at T5. (a) ROC curve demonstrating the ability of these microbes to discriminate between success and failure outcomes, with AUC values indicating predictive accuracy. Microbes included: *Campylobacter showae*, *Fusobacterium polymorphum*, *Lachnoanaerobaculum* sp., *Leptothrix* sp., *Rothia dentocariosa*, *Selenomonas* sp. and *Streptococcus sanguinis*. (b) Boxplots showing the relative abundance of differentially abundant microbes at T5.

Differentially abundant taxa, including both FDR‐significant and *p*‐value level associations identified at T2 and T5, were evaluated for predictive performance using ROC analysis. At T5, several taxa exhibited moderate predictive ability (AUC > 0.70), including *R. dentocariosa* (AUC = 0.753, sensitivity = 0.714, specificity = 0.864), *Leptothrix* sp. (AUC = 0.792, sensitivity = 0.857, specificity = 0.682), *F. polymorphum* (AUC = 0.753, sensitivity = 0.857, specificity = 0.682), *C. showae* (AUC = 0.74, sensitivity = 0.571, specificity = 0.909), *Lachnoanaerobaculum* sp. (AUC = 0.701, sensitivity = 0.571, specificity = 0.909), *Selenomonas* sp. (AUC = 0.818, sensitivity = 0.714, specificity = 0.909) and *S. sanguinis* (AUC = 0.701, sensitivity = 1, specificity = 0.454) (Figure 4a). Also, the log_10_‐transformed abundances of these microbes across samples are shown as box plots in Figure 4b, comparing treatment success and failure groups. Other taxa showed low predictive power (Table S5). In contrast, microbes enriched at T2 did not demonstrate strong predictive ability, indicating that taxa associated with T5 are more informative for discriminating between successful and failed treatment outcomes.

### 3.6 Selective Persistence of Key Microbial Taxa Shapes Treatment Success and Failure

Microbial persistence was assessed in paired samples from T1 → T5 and T2 → T5 in 25 patients (18 successes and 7 failures). Among 196 species analysed from T1 → T5, only three showed statistically significant differences in persistence. *F. polymorphum* persisted in all failure cases (7/7) and success cases (8/18). *R. dentocariosa* showed higher persistence in success cases (16/18) than in failures (3/7), whereas *C. showae* persisted more in failures (4/7) than in successes (2/18). Between T2 and T5 (150 species), *R. dentocariosa* (14/18 in success and 1/7 in failure) showed greater persistence in successful outcomes. Conversely, *Selenomonas* sp. (3/7 in failure and 0/18 in success) and *C. showae* (4/7 in failure and 2/18 in success) showed greater persistence in failure cases (*p* < 0.05). These patterns suggest persistence of specific taxa, including *F. polymorphum*, *C. showae* and *Selenomona*s sp., across treatment time points, with *F*. *polymorphum* detected from T1 → T5 and *C. showae* and *Selenomonas* sp. from T2 → T5 following intracanal procedures. Conversely, greater persistence of *R. dentocariosa* in successful cases suggests distinct microbial persistence patterns across treatment outcomes. A summary of microbial persistence across the analysed time points is provided in Table S6.

### 3.7 Functional Pathway Associated With Success and Failure Outcomes

**FIGURE 5:**
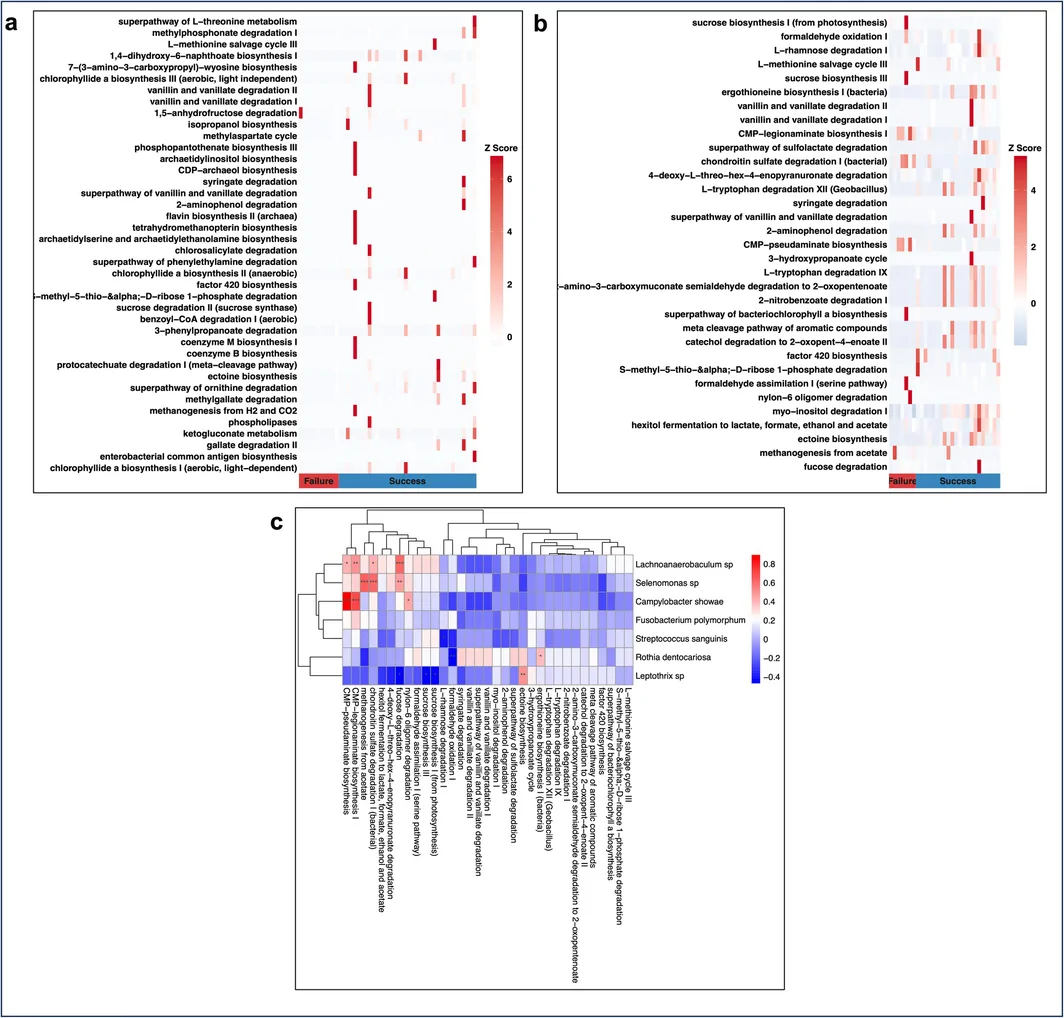
Functional prediction of microbial communities and their associations with metabolic pathways across treatment outcomes. (a, b) Heatmaps show significantly enriched MetaCyc metabolic pathways (adjusted *p* < 0.05) at T2 (root canal before debridement) and T5 (after calcium hydroxide dressing) between treatment success and failure groups. Pathway abundance values were *z*‐score‐normalized, with red indicating higher and blue indicating lower relative functional potential. (c) Spearman correlation heatmap illustrates associations between T5‐enriched microbes and predicted metabolic pathways. Red indicates positive correlations, blue indicates negative correlations, and asterisks denote statistically significant correlations (*p* < 0.05).

Functional profiling revealed differences in metabolic potentials of microbial communities associated with treatment outcomes, with an adjusted *p*‐value < 0.05. At T2, the failure group showed enrichment of 1,5‐anhydrofructose degradation, while the success group was enriched in 39 pathways including flavin biosynthesis II (archaea), methanogenesis from H2 and CO_2_, chlorophyllide a biosynthesis (I, II, III), coenzyme B and M biosynthesis, sucrose degradation II (sucrose synthase), L‐methionine salvage cycle III, benzoyl‐CoA degradation I (aerobic), and vanillin/vanillate degradation and ectoine biosynthesis were significantly enriched. At T5, nine pathways including sucrose biosynthesis III, sucrose biosynthesis I from photosynthesis, formaldehyde assimilation (serine pathway), CMP‐pseudaminate biosynthesis, and bacteriochlorophyll a biosynthesis were enriched in failure, while 24 pathways including ectoine, ergothioneine biosynthesis, L‐methionine salvage cycle III, myo‐inositol degradation, L‐tryptophan and L‐rhamnose degradation, catechol degradation, vanillin and vanillate degradation I & II were enriched in successes (depicted in Figure 5a,b).

Spearman's correlation analysis revealed associations between T5 enriched taxa and predicted metabolic pathways. *Lachnoaerobaculum* sp., *C. showae* and *Selenomonas* sp. were positively correlated with several pathways, including fucose degradation, CMP‐pseudaminate biosynthesis, CMP‐legionaminate biosynthesis I, chondroitin sulfate degradation I (bacterial), nylon‐6 oligomer degradation and methanogenesis from acetate, suggesting that higher abundance of these microbes may be linked to increased activity in these metabolic processes. Conversely, *R. dentocariosa* showed a negative correlation with formaldehyde oxidation I and a positive correlation with ergothioneine biosynthesis I (bacteria), whereas *Leptothrix* sp. was negatively associated with fucose degradation, sucrose biosynthesis III, sucrose biosynthesis I (from photosynthesis) and positively with ectoine biosynthesis. Additionally, *S. sanguinis* was negatively correlated with L‐rhamnose degradation I (Figure 5c). These results indicate that the T5‐enriched taxa may influence treatment outcomes by modulating predicted microbial metabolic pathways; however, experimental validation would be required to confirm these functional associations.

## 4 Discussion

The present study provides an overview of microbial dynamics during RET, suggesting that the root canal microbiota undergoes significant shifts throughout treatment. There was a significant reduction in microbial species richness following initial debridement, reflecting the immediate impact of disinfection. Alpha diversity tended to be higher in failed cases, although not significant. Following intracanal dressing, taxa, such as *R. dentocariosa* and *Leptothrix* sp., were enriched in successful outcomes, showing moderate potential discriminative performance (AUC = 0.753–0.792) while *F. polymorphum*, *C. showae* , *Selenomonas* sp., *Lachnoanaerobaculum* sp. and *S. sanguinis* , were significantly enriched in failure cases (AUC = 0.701–0.818). These findings suggest potential microbial signatures associated with outcomes that warrant further validation in independent cohorts. Predicted functional profiling revealed differential enrichment of metabolic pathways between successful and failed treatment groups.

The taxonomic composition observed in this study was broadly consistent with previous reports of root canal microbiota, with Actinomycetota, Bacillota, Bacteroidota, Fusobacteriota and Pseudomonadota representing the dominant phyla in intracanal samples (Wikström et al. 2024). Overall, changes in relative abundance across treatment time points, particularly following debridement and intracanal dressing, in line with previous studies reporting that endodontic treatment can influence microbial community composition (Fouad et al. 2022). However, several dominant phyla persisted across time points, and thus, interpretation based on taxonomic composition alone remains limited. In contrast, external surface samples showed relatively similar profiles, differing from intracanal samples, supporting a distinction between external and root canal microbial communities. Although some differences in microbial composition were observed between successful and failed cases, these patterns were not consistent across time points and did not clearly distinguish treatment outcomes. Overall, these findings suggest that taxonomic composition alone may have limited value in explaining differences in treatment outcomes.

Alpha diversity analyses also revealed significant differences between external tooth surfaces and intracanal communities, with higher species richness and diversity in external samples. Within intracanal samples, diversity varied across treatment time points, with a reduction in species richness from T2 to T3 followed by an increase at T5. Shannon and InvSimpson indices remained relatively stable, suggesting limited variation in community evenness relative to richness. When stratified by treatment outcome, alpha diversity showed no consistent or statistically significant differences between successful and failed cases across time points. A significant difference was observed only at T3, where successes showed higher Chao1 richness and Observed species; however, this pattern was not maintained at other time points, and a non‐significant reversal was observed at T5. Overall, alpha diversity was not consistently associated with differences between treatment outcomes. Beta diversity analysis showed that microbial community composition was significantly associated with time points rather than treatment outcome, with no clear separation between groups. These findings suggest that while intracanal microbial communities undergo temporal changes during treatment, these changes are not consistently associated with treatment outcomes. This supports further investigation through differential abundance analysis to identify outcome‐associated taxa.

Differential abundance analysis (Table S4) identified taxa associated with the initial infected state, including *P. endodontalis* and *F*. *vincentii*, particularly in failed cases, consistent with the polymicrobial and anaerobic nature of primary endodontic infections (Gomes et al. 2007; Siqueira et al. 2024). Following intracanal dressing, distinct microbial profiles were observed between outcome groups, suggesting that treatment outcome may be influenced not only by overall bacterial reduction but also by selective persistence of specific taxa. Several taxa associated with oral dysbiosis and persistent endodontic infections, including *F. polymorphum*, *C. showae* , *Lachnoanaerobaculum* sp., *S. sanguinis* and *Selenomonas* sp. were more frequently detected in failed cases at T5. Persistence analysis further suggested that certain taxa were more resilient to intracanal treatment, suggesting their continued presence after treatment. Whether these taxa contribute to treatment failure or simply reflect a treatment‐resistant microbial community remains unclear.

The detection of taxa such as *C. showae* and *Selenomona*s sp. in failed cases aligns with earlier reports linking these taxa to persistent endodontic infections (Tronstad and Sunde 2003) and post‐treatment apical periodontitis (Anderson et al. 2012). *F. nucleatum* subsp. *polymorphum*, a well‐known oral bacterium associated with extra‐oral translocation and systemic inflammation (Tomás‐Cortázar et al. 2018; Karpathy et al. 2007; Han and Wang 2013). *Lachnoanaerobaculum* sp. has also been identified in endodontic infections (Sassone et al. 2012). In contrast, *R. dentocariosa* and *Leptothrix* sp. were enriched in successful cases. *R. dentocariosa* is commonly associated with health‐associated oral microbiota rather than active endodontic infections (Colombo et al. 2009). Although the role of *Leptothrix* sp. in the root canal microbiota remains poorly understood, highlighting the presence of undercharacterized taxa in post‐treatment microbial communities. The moderate ROC performance of several T5‐associated taxa further suggests that post‐treatment microbial composition may have discriminatory potential for distinguishing treatment outcomes, supporting the possibility that persistence of infection‐associated taxa may reflect incomplete microbial transition after treatment. However, these observations should be considered exploratory and require validation in larger cohorts. Overall, these findings suggest that treatment success is associated with a shift in intracanal microbial composition towards a less disease‐associated community profile. Although the persistence of specific anaerobic taxa in failed cases suggests that the microbial community did not fully shift after treatment, and still retained infection‐associated microbes. However, given the limitations of 16S rRNA‐based profiling, these taxa should be interpreted as potential markers rather than causal drivers of treatment outcomes, and their mechanistic roles in RET remain to be established.

Functional profiling based on PICRUSt2 suggested differences in predicted metabolic potentials of microbial communities associated with treatment outcomes, particularly at T5. It is important to note, however, that these findings represent inferred metabolic potential based on taxonomic composition rather than direct measurements of microbial activity. Our analysis suggested that failure‐associated taxa were predicted to be linked with metabolic pathways such as fucose degradation, CMP‐pseudaminate biosynthesis and methanogenesis. These pathways are associated with nutrient acquisition, host–microbe interactions, and microbial persistence, including virulence‐associated glycan structures (Vibhute et al. 2021; Ng et al. 2013; Schoenhofen et al. 2009). Conversely, success‐associated taxa were associated with pathways such as biosynthesis of ergothioneine and ectoine, suggesting potential stress‐adaptive or protective functions, since this compound is known to act as a microbial stress protectant with antioxidant or osmoprotective properties (Cumming et al. 2018; Sato et al. 2025; Richter et al. 2019). These inferred functional differences suggest that microbial metabolic capacity may influence treatment outcomes, though experimental confirmation (e.g., metabolomics) would be required to confirm these predictions.

Supporting this, literature‐based evidence indicates that failure‐associated taxa such as *Fusobacterium* sp. and *Selenomonas* sp. have been linked to proteolytic metabolism and short‐chain fatty acid production, features commonly observed in anaerobic microbial communities (Sakanaka et al. 2022; Dahlstrand Rudin et al. 2021; Leonov et al. 2023). Conversely, *R. dentocariosa* has been associated with nitrate‐reduction pathways that may contribute to pH regulation and suppression of acidogenic and pathogenic species in oral environments (Rosier, Buetas, et al. 2020; Rosier, Moya‐Gonzalvez, et al. 2020). Overall, integration of PICRUSt2‐derived functional predictions and literature‐based functional evidence provides biological context for the observed microbial differences, although these findings remain inferential and require experimental validation.

### 4.1 Limitations and Future Research of the Study

One of the main shortcomings of this study was its relatively small sample size, particularly when stratifying samples based on success and failure cases at individual time points, which reduced statistical power for subgroup analyses. Although taxonomic analyses and ROC assessments were performed to identify taxa associated with treatment outcomes, larger cohorts would strengthen the reliability and generalizability of these findings. In addition, 16S rRNA gene sequencing provides taxonomic resolution primarily at the genus level, while the use of eHOMD enabled improved species‐level assignment for oral‐associated taxa; however, strain‐level variation and direct microbial activity were not captured. Functional predictions based on computational inference further reflect potential metabolic capacity rather than experimentally validated activity. Despite these limitations, the study provides clinically relevant insights into temporal microbial shifts during RET and offers a baseline for future biomarker‐driven research.

## 5 Conclusion

In summary, this study demonstrates that regenerative endodontic treatment induces significant changes in the root canal microbiome that are associated with treatment outcomes. Differentially abundant microbial species enriched following intracanal dressing, including *F. polymorphum*, *C. showae* , *Selenomonas* sp., *Lachnoanaerobaculum* sp. and *S. sanguinis* , were linked to treatment failure, whereas *R. dentocariosa* and *Leptothrix* sp. were enriched in treatment success. These taxa exhibited moderate discriminative potential between outcomes, highlighting their potential as predictive biomarkers. These findings highlight the predictive potential of the post‐treatment microbiome and suggest that its monitoring could improve regenerative endodontic outcomes.

## Author Contributions

Conceptualisation: N.R.V., A.W. and M.B. Methodology: L.D.N., L.M., A.W. and N.R.V. Formal analysis: L.D.N. and L.M. Investigation: L.D.N., L.M. and O.R. Data curation: L.D.N., L.M., O.R. and N.R.V. Visualization: L.D.N., L.M. and O.R. Laboratory investigation: P.Q. and O.R. Funding acquisition: M.B. and N.R.V. Project administration: A.W. and N.R.V. Supervision: M.B. and N.R.V. Writing – original draft: L.D.N., A.W., P.Q., O.R. and N.R.V. Writing – review and editing: All authors.

## Funding

This work was supported by the Region of Västerbotten (Sweden) via TUA grant number 977100, ALF grant number RV‐967705 and Kempestiftelserna Kempe JCSMK23‐0158.

## Conflicts of Interest

The authors declare no conflicts of interest.

## Supporting information


**Figure S1:** Taxonomic composition of microbial communities across time points and treatment outcomes. (a) Sample‐wise stacked bar plot depicting the relative abundance of the top phyla across all samples from T1 to T5. Each bar represents an individual sample ordered by time point; low‐abundance taxa are grouped as ‘Other’. (b) Sample‐wise stacked bar plots showing the relative abundance of the top families across all samples from T1 to T5. Each bar represents an individual sample ordered by time point; remaining taxa are aggregated as ‘Other’. (c) Sample‐wise stacked bar plots at the phylum level stratified by treatment outcome (success vs. failure). Samples are organized across time points (T1–T5), with each bar representing an individual sample. Low‐abundance taxa are grouped as ‘Other’.

**Table S1:** Mean relative abundances (%) of microbial taxa at the phylum, family and genus levels across all time points.
**Table S2:** Pairwise comparisons of alpha diversity metrics (Chao1, Observed, Shannon and InvSimpson) across time points (T1–T5).
**Table S3:** PERMANOVA analysis of the effects of demographic and clinical variables on beta diversity at time points T2 and T5.
**Table S4:** Differential abundance of microbial taxa associated with outcomes across T2 and T5 (ANCOM‐BC2, MaAsLin2 and LEfSe analyses).
**Table S5:** Receiver Operating Characteristic (ROC) analysis of key microbial taxa for discriminating treatment outcomes.
**Table S6:** Persistence and resilience of microbial species from T1 and T2 samples to T5. The table lists species detected at T1 or T2 and retained at T5. Count_S_P and count_S_NP represent persistent and non‐persistent species in successful cases, whereas count_F_P and count_F_NP represent persistent and non‐persistent species in failure cases. Odds Ratio (OR) indicates the likelihood of persistence, and *p*‐value denotes statistical significance. ‘Inf’ indicates species found exclusively in one group.

**Data S1:** Comparison of demographic and clinical characteristics between treatment outcomes, success and failure.
**Data S2:** Summary of sequence processing for each sample. The table shows the number of raw reads per sample (input), reads passing quality filtering (filtered), reads successfully denoised (denoised), reads successfully merged (merged) and reads retained after chimera removal (non‐chimeric). Percentages indicate the proportion of input reads retained at each step.
**Data S3:** Taxonomic assignment of all identified amplicon sequence variants (ASVs). This table lists each feature ID along with its assigned taxon, assignment confidence, and full taxonomic hierarchy from kingdom to species.
**Data S4:** Associations between demographic and clinical variables and alpha diversity metrics (Chao1, Observed, Shannon and InvSimpson) across sampling time points (T2, T5).

## Data Availability

The sequencing data generated in this study are available in European Nucleotide Archive (ENA) under accession number PRJEB111893 while the previously published data used from our research group integrated into this study are available under accession number PRJEB61279.
