# Supplementary material for: Distinct Microbiota and Functional Pathway Profiles Define Success and Failure in Regenerative Endodontic Treatment
Source: Int Endod J. 2026 Jun 23;59(10):2223–37. doi: 10.1111/iej.70205 (PMC13569218; doi:10.1111/iej.70205)
Supplement: Supplementary file 1 [file IEJ-59-2223-s001.docx]

**Supplementary Table 1:** Mean relative abundances (%) of microbial taxa at the phylum, family, and genus levels across all time points.

| **Time Points** | **Phylum** | **Mean Abundance** | **Time Points** | **Family** | **Mean Abundance** | **Time Points** | **Genus** | **Mean Abundance** |
| --- | --- | --- | --- | --- | --- | --- | --- | --- |
| T1 | Bacillota | 0.2540 | T5 | Other | 0.3291 | T4 | *Actinomyces* | 0.1368 |
| T1 | Bacteroidota | 0.0568 | T3 | Pseudomonadaceae | 0.1256 | T1 | *Actinomyces* | 0.0860 |
| T5 | Bacteroidota | 0.0850 | T4 | Pseudomonadaceae | 0.0183 | T5 | *Actinomyces* | 0.0851 |
| T3 | Cyanobacteriota | 0.0130 | T2 | Veillonellaceae | 0.0539 | T3 | *Actinomyces* | 0.0274 |
| T2 | Patescibacteria | 0.0072 | T4 | Micrococcaceae | 0.0870 | T2 | *Actinomyces* | 0.0151 |
| T1 | Mycoplasmatota | 0.0000 | T5 | Veillonellaceae | 0.0258 | T1 | *Capnocytophaga* | 0.0186 |
| T3 | Other | 0.0000 | T4 | Fusobacteriaceae | 0.0433 | T3 | *Capnocytophaga* | 0.0161 |
| T1 | Other | 0.0001 | T1 | Veillonellaceae | 0.0269 | T4 | *Capnocytophaga* | 0.0214 |
| T2 | Pseudomonadota | 0.1137 | T1 | Actinomycetaceae | 0.0947 | T2 | *Capnocytophaga* | 0.0435 |
| T5 | Synergistota | 0.0004 | T2 | Micrococcaceae | 0.0125 | T5 | *Capnocytophaga* | 0.0495 |
| T4 | Bacteroidota | 0.0707 | T1 | Streptococcaceae | 0.1505 | T4 | *Corynebacterium* | 0.0877 |
| T5 | Fusobacteriota | 0.0623 | T5 | Prevotellaceae | 0.0180 | T5 | *Corynebacterium* | 0.0817 |
| T4 | Pseudomonadota | 0.1897 | T3 | Enterobacteriaceae | 0.2060 | T1 | *Corynebacterium* | 0.0908 |
| T2 | Synergistota | 0.0161 | T4 | Corynebacteriaceae | 0.0874 | T2 | *Corynebacterium* | 0.0224 |
| T3 | Synergistota | 0.0098 | T3 | Streptococcaceae | 0.0502 | T3 | *Corynebacterium* | 0.0253 |
| T3 | Actinomycetota | 0.1247 | T5 | Micrococcaceae | 0.0674 | T3 | *Enterobacter* | 0.1246 |
| T2 | Mycoplasmatota | 0.0009 | T1 | Micrococcaceae | 0.0806 | T2 | *Enterobacter* | 0.0000 |
| T4 | Other | 0.0000 | T3 | Corynebacteriaceae | 0.0248 | T1 | *Enterobacter* | 0.0001 |
| T5 | Patescibacteria | 0.0158 | T2 | Actinomycetaceae | 0.0176 | T5 | *Enterobacter* | 0.0641 |
| T4 | Spirochaetota | 0.0028 | T4 | Prevotellaceae | 0.0290 | T4 | *Enterobacter* | 0.0320 |
| T2 | Bacteroidota | 0.2748 | T5 | Enterobacteriaceae | 0.0951 | T2 | *Fusobacterium* | 0.0982 |
| T3 | Fusobacteriota | 0.0621 | T1 | Pseudomonadaceae | 0.0599 | T3 | *Fusobacterium* | 0.0496 |
| T5 | Other | 0.0000 | T1 | Enterobacteriaceae | 0.0032 | T5 | *Fusobacterium* | 0.0328 |
| T1 | Synergistota | 0.0035 | T3 | Neisseriaceae | 0.0314 | T4 | *Fusobacterium* | 0.0434 |
| T4 | Synergistota | 0.0003 | T3 | Prevotellaceae | 0.0271 | T1 | *Fusobacterium* | 0.0240 |
| T1 | Cyanobacteriota | 0.0195 | T3 | Actinomycetaceae | 0.0328 | T4 | *Neisseria* | 0.0649 |
| T2 | Cyanobacteriota | 0.0013 | T5 | Corynebacteriaceae | 0.0809 | T2 | *Neisseria* | 0.0095 |
| T4 | Fusobacteriota | 0.0984 | T4 | Actinomycetaceae | 0.1520 | T1 | *Neisseria* | 0.0901 |
| T3 | Spirochaetota | 0.0052 | T4 | Enterobacteriaceae | 0.0501 | T3 | *Neisseria* | 0.0245 |
| T5 | Spirochaetota | 0.0022 | T1 | Prevotellaceae | 0.0126 | T5 | *Neisseria* | 0.0557 |
| T4 | Actinomycetota | 0.3440 | T2 | Other | 0.6520 | T1 | Other | 0.3782 |
| T2 | Bacillota | 0.3550 | T2 | Fusobacteriaceae | 0.0982 | T2 | Other | 0.7123 |
| T5 | Pseudomonadota | 0.2889 | T1 | Fusobacteriaceae | 0.0240 | T5 | Other | 0.3529 |
| T3 | Pseudomonadota | 0.4500 | T4 | Neisseriaceae | 0.0743 | T3 | Other | 0.5278 |
| T1 | Pseudomonadota | 0.3277 | T3 | Other | 0.4037 | T4 | Other | 0.3509 |
| T5 | Bacillota | 0.2583 | T3 | Micrococcaceae | 0.0210 | T5 | *Pseudomonas* | 0.0872 |
| T3 | Bacillota | 0.2123 | T2 | Prevotellaceae | 0.0724 | T3 | *Pseudomonas* | 0.1274 |
| T4 | Mycoplasmatota | 0.0000 | T2 | Pseudomonadaceae | 0.0249 | T4 | *Pseudomonas* | 0.0183 |
| T1 | Patescibacteria | 0.0194 | T5 | Fusobacteriaceae | 0.0325 | T1 | *Pseudomonas* | 0.0591 |
| T2 | Spirochaetota | 0.0439 | T1 | Corynebacteriaceae | 0.0907 | T2 | *Pseudomonas* | 0.0248 |
| T5 | Cyanobacteriota | 0.0051 | T2 | Neisseriaceae | 0.0150 | T5 | *Rothia* | 0.0562 |
| T1 | Fusobacteriota | 0.0450 | T4 | Streptococcaceae | 0.1218 | T4 | *Rothia* | 0.0853 |
| T3 | Mycoplasmatota | 0.0000 | T4 | Veillonellaceae | 0.0406 | T3 | *Rothia* | 0.0090 |
| T2 | Other | 0.0013 | T5 | Pseudomonadaceae | 0.0864 | T2 | *Rothia* | 0.0110 |
| T4 | Patescibacteria | 0.0398 | T5 | Streptococcaceae | 0.1123 | T1 | *Rothia* | 0.0783 |
| T2 | Actinomycetota | 0.0753 | T5 | Actinomycetaceae | 0.0918 | T2 | *Streptococcus* | 0.0298 |
| T5 | Actinomycetota | 0.2821 | T2 | Corynebacteriaceae | 0.0224 | T5 | *Streptococcus* | 0.1119 |
| T1 | Actinomycetota | 0.2708 | T1 | Other | 0.3625 | T1 | *Streptococcus* | 0.1501 |
| T4 | Bacillota | 0.2527 | T4 | Other | 0.2961 | T4 | *Streptococcus* | 0.1222 |
| T3 | Bacteroidota | 0.1204 | T5 | Neisseriaceae | 0.0605 | T3 | *Streptococcus* | 0.0499 |
| T4 | Cyanobacteriota | 0.0015 | T3 | Veillonellaceae | 0.0288 | T1 | *Veillonella* | 0.0246 |
| T2 | Fusobacteriota | 0.1105 | T1 | Neisseriaceae | 0.0944 | T2 | *Veillonella* | 0.0333 |
| T5 | Mycoplasmatota | 0.0000 | T2 | Enterobacteriaceae | 0.0007 | T5 | *Veillonella* | 0.0229 |
| T3 | Patescibacteria | 0.0026 | T3 | Fusobacteriaceae | 0.0487 | T3 | *Veillonella* | 0.0185 |
| T1 | Spirochaetota | 0.0031 | T2 | Streptococcaceae | 0.0306 | T4 | *Veillonella* | 0.0370 |

**Supplementary Table 2**: Pairwise comparisons of alpha diversity metrics (Chao1, Observed, Shannon, InvSimpson) across time points (T1–T5).

| **Metric** | **group1** | **group2** | **Z** | **P.adj** | **label** |
| --- | --- | --- | --- | --- | --- |
| Chao1 | T1 | T2 | 2.873502 | 0.004059 | ** |
| Chao1 | T1 | T3 | 4.916631 | 2.20E-06 | *** |
| Chao1 | T2 | T3 | 2.556877 | 0.007544 | ** |
| Chao1 | T1 | T4 | -1.9815 | 0.026408 | * |
| Chao1 | T2 | T4 | -4.407543 | 1.74E-05 | *** |
| Chao1 | T3 | T4 | -6.06716 | 6.51E-09 | *** |
| Chao1 | T1 | T5 | 2.646643 | 0.006775 | ** |
| Chao1 | T3 | T5 | -1.996538 | 0.028672 | * |
| Chao1 | T4 | T5 | 4.070622 | 5.86E-05 | *** |
| Observed | T1 | T2 | 3.029459 | 0.00245 | ** |
| Observed | T1 | T3 | 5.326918 | 2.50E-07 | *** |
| Observed | T2 | T3 | 2.840469 | 0.003754 | ** |
| Observed | T1 | T4 | -1.799253 | 0.039988 | * |
| Observed | T2 | T4 | -4.354183 | 2.23E-05 | *** |
| Observed | T3 | T4 | -6.26773 | 1.83E-09 | *** |
| Observed | T1 | T5 | 2.637002 | 0.005974 | ** |
| Observed | T3 | T5 | -2.36588 | 0.011242 | * |
| Observed | T4 | T5 | 3.90185 | 0.000119 | *** |
| Shannon | T1 | T2 | 3.779311 | 0.000262 | *** |
| Shannon | T1 | T4 | -2.67997 | 0.007363 | ** |
| Shannon | T2 | T4 | -5.871481 | 2.16E-08 | *** |
| Shannon | T3 | T4 | -3.738419 | 0.000231 | *** |
| Shannon | T2 | T5 | -3.790937 | 0.000375 | *** |
| Shannon | T3 | T5 | -1.925922 | 0.045095 | * |
| Shannon | T4 | T5 | 1.812497 | 0.049935 | * |
| InvSimpson | T1 | T2 | 3.141383 | 0.002803 | ** |
| InvSimpson | T2 | T3 | -2.561154 | 0.010433 | * |
| InvSimpson | T1 | T4 | -2.651765 | 0.010009 | * |
| InvSimpson | T2 | T4 | -5.308641 | 5.52E-07 | *** |
| InvSimpson | T3 | T4 | -2.393514 | 0.013907 | * |
| InvSimpson | T2 | T5 | -3.921612 | 0.00022 | *** |

**Supplementary Table 3**: PERMANOVA analysis of the effects of demographic and clinical variables on beta diversity at time points T2 and T5.

| **T2** | | | | | |
| --- | --- | --- | --- | --- | --- |
|  | **Df** | **SumOfSqs** | **R^2^** | **F** | **Pr(>F)** |
| **Outcomes** | 1 | 0.4084 | 0.01596 | 0.878 | 0.7951 |
| **AGE** | 1 | 0.4689 | 0.01832 | 1.0081 | 0.445 |
| **SEX** | 1 | 0.4182 | 0.01634 | 0.8992 | 0.7414 |
| **PAI** | 3 | 1.4651 | 0.05725 | 1.05 | 0.2551 |
| **ABSCESS** | 1 | 0.5473 | 0.02139 | 1.1767 | 0.1119 |
| **TRAUMA_TYPE_BOTH** | 2 | 1.0187 | 0.03981 | 1.0951 | 0.1646 |
| **ROOT_DEV_STAGE** | 4 | 1.986 | 0.0776 | 1.0675 | 0.1746 |
| **BONE_DIAGNOSIS** | 2 | 0.9082 | 0.03549 | 0.9763 | 0.5925 |
| **INFL_RESORPTION** | 1 | 0.4653 | 0.01818 | 1.0004 | 0.4578 |
| **MOBILITY** | 1 | 0.4114 | 0.01608 | 0.8846 | 0.7946 |
| **TENDERNESS_TO_PALPATION** | 1 | 0.3198 | 0.01249 | 0.6875 | 0.9858 |
| **SINUS_TRACT** | 1 | 0.4297 | 0.01679 | 0.924 | 0.678 |
| **Residual** | 36 | 16.7442 | 0.65429 |  |  |
| **Total** | 55 | 25.5916 | 1.00000 |  |  |

| **T5** | | | | | |
| --- | --- | --- | --- | --- | --- |
|  | **Df** | **SumOfSqs** | **R^2^** | **F** | **Pr(>F)** |
| **Outcomes** | 1 | 0.4411 | 0.04012 | 1.1325 | 0.2455 |
| **AGE** | 1 | 0.5346 | 0.04862 | 1.3725 | 0.0542 |
| **SEX** | 1 | 0.2705 | 0.0246 | 0.6945 | 0.956 |
| **PAI** | 2 | 0.7013 | 0.06378 | 0.9003 | 0.7326 |
| **ABSCESS** | 1 | 0.2798 | 0.02545 | 0.7184 | 0.9379 |
| **TRAUMA_TYPE_BOTH** | 1 | 0.4019 | 0.03655 | 1.0318 | 0.4006 |
| **ROOT_DEV_STAGE** | 2 | 0.7869 | 0.07156 | 1.0101 | 0.447 |
| **BONE_DIAGNOSIS** | 1 | 0.3466 | 0.03152 | 0.8898 | 0.6898 |
| **INFL_RESORPTION** | 1 | 0.3806 | 0.03462 | 0.9772 | 0.5006 |
| **MOBILITY** | 1 | 0.394 | 0.03584 | 1.0116 | 0.4545 |
| **TENDERNESS_TO_PALPATION** | 1 | 0.3693 | 0.03359 | 0.9482 | 0.572 |
| **SINUS_TRACT** | 1 | 0.3121 | 0.02838 | 0.8013 | 0.8378 |
| **Residual** | 13 | 5.0636 | 0.46050 |  |  |
| **Total** | 28 | 10.9958 | 1.00000 |  |  |

Df = degrees of freedom; SumOfSqs = sum of squares; R² = proportion of variance explained; F = F-statistic; Pr(>F) = p-value

**Supplementary Table 4**: Differential abundance of microbial taxa associated with outcomes across T2 and T5 (ANCOM-BC2, MaAsLin2, and LEfSe analyses).

|  |  | **ANCOM-BC2** | | | **MAASLIN2** | | | **LEfSe** | | |
| --- | --- | --- | --- | --- | --- | --- | --- | --- | --- | --- |
| **T2** | **taxon** | **lfc** | **pval** | **qval** | **coef** | **pval** | **qval** | **enrich_group** | **ef_lda** | **pval** |
|  | *Slackia exigua* | 0.9710 | 0.0200 | **0.0633** | na | na | na | na | na | na |
|  | *Staphylococcus* sp. | 0.9088 | 0.0416 | 0.1134 | na | na | na | na | na | na |
|  | *Porphyromonas endodontalis* | -1.3559 | 0.0156 | **0.0512** | na | na | na | na | na | na |
|  | *Tannerella forsythia* | 1.7816 | 0.0254 | **0.0773** | na | na | na | na | na | na |
|  | *Fusobacterium vincentii* | -1.2439 | 0.0270 | **0.0791** | na | na | na | na | na | na |
|  | *Lautropia mirabilis* | -1.1137 | 0.0402 | 0.1134 | na | na | na | na | na | na |
|  | *Staphylococcus warneri* | na | na | na | -1.2549 | 0.0033 | 0.3689 | na | na | na |
|  | *Cardiobacterium valvarum* | na | na | na | -1.354 | 0.0203 | 0.6967 | na | na | na |
|  | *Granulicatella adiacens* | na | na | na | -3.3513 | 0.0163 | 0.6967 | na | na | na |
|  | *Hoylesella* sp. HMT 317 | na | na | na | -2.0523 | 0.0247 | 0.6967 | na | na | na |
|  | *Lancefieldella* sp. HMT 199 | na | na | na | -1.8294 | 0.0134 | 0.6967 | na | na | na |
|  | *Porphyromonas catoniae* | na | na | na | -2.6588 | 0.0207 | 0.6967 | na | na | na |
|  | *Lachnoanaerobaculum sp* | na | na | na | -1.8222 | 0.0326 | 0.7873 | na | na | na |
|  | *Eikenella corrodens* | na | na | na | -2.6556 | 0.0389 | 0.8771 | na | na | na |
| **T5** | *Actinomyces naeslundii* | -1.6823 | **0.0143** | **0.0720** | na | na | na | na | na | na |
|  | *Lancefieldella parvula* | 1.9166 | 0.0467 | 0.1557 | na | na | na | na | na | na |
|  | *Streptococcus sanguinis* | -1.1245 | 0.0288 | 0.1153 | na | na | na | na | na | na |
|  | *Lachnoanaerobaculum* sp. | -1.2602 | **0.0144** | **0.0720** | -1.5148 | 0.0376 | 0.5502 |  |  |  |
|  | *Neisseria elongata* | -1.2548 | 0.0271 | 0.1153 | na | na | na | na | na | na |
|  | *Escherichia coli* | -1.3332 | 0.0461 | 0.1557 | na | na | na | na | na | na |
|  | *Cardiobacterium valvarum* | na | na | na | -1.6174 | 0.0038 | 0.3568 | na | na | na |
|  | *Selenomonas* sp. | na | na | na | -3.9655 | 0.0019 | 0.3568 | na | na | na |
|  | *Lachnoanaerobaculum saburreum* | na | na | na | -2.4426 | 0.0062 | 0.3897 | Failure | **3.0229** | **0.0248** |
|  | *Campylobacter showae* | na | na | na | -2.4630 | 0.0118 | 0.3918 | Failure | **3.1160** | **0.0077** |
|  | *Comamonas testosteroni* | na | na | na | -1.4681 | 0.0108 | 0.3918 | na | na | na |
|  | *Leptotrichia* sp. HMT 417 | na | na | na | -1.9702 | 0.0131 | 0.3918 | na | na | na |
|  | *Rothia dentocariosa* | na | na | na | 5.0443 | 0.0144 | 0.3918 | Success | **4.4696** | **0.0463** |
|  | *Aggregatibacter* sp. HMT 898 | na | na | na | -0.5545 | 0.0207 | 0.4062 | na | na | na |
|  | *Leptotrichia hofstadii* | na | na | na | -1.4671 | 0.0214 | 0.4062 | na | na | na |
|  | *Segatella oris* | na | na | na | -3.3159 | 0.0191 | 0.4062 | na | na | na |
|  | *Leptothrix* sp. | na | na | na | 3.6610 | 0.0266 | 0.4219 | Success | **4.0229** | **0.0323** |
|  | *Prevotella veroralis* | na | na | na | -0.8971 | 0.0253 | 0.4219 | na | na | na |
|  | *Hoylesella* sp. HMT 317 | na | na | na | -2.9680 | 0.0424 | 0.5758 | na | na | na |
|  | *Arachnia propionica* | na | na | na | -2.8395 | 0.0465 | 0.5893 | Failure | **4.1394** | **0.0302** |
|  | *Fusobacterium polymorphum* | na | na | na | na | na | na | Failure | **4.1119** | **0.0424** |

**LFC**: logfold change from ANCOM-BC output, **Coef**: the model coefficient value (effect size) in MaAsLin2 output, **ef_lda**: Effect Size for Linear Discriminant Analysis for LEfSe output

**Supplementary Table 5:** ROC analysis of key microbial taxa for discriminating treatment outcomes.

| **Feature** | **Area under the curve value** | **95% CI** | **Sensitivity** | **Specificity** |
| --- | --- | --- | --- | --- |
| ***T2 enriched microbes*** | | | | |
| *Slackia exigua* | 0.477 | 0.322-0.632 | 0.364 | 0.658 |
| *Staphylococcus* sp. | 0.486 | 0.299-0.672 | 0.545 | 0.579 |
| *Porphyromonas endodontalis* | 0.502 | 0.337-0.668 | 0.182 | 0.921 |
| *Tannerella forsythia* | 0.541 | 0.39-0.692 | 0.273 | 0.816 |
| *Fusobacterium vincentii* | 0.505 | 0.337-0.673 | 0.182 | 0.921 |
| *Lautropia mirabilis* | 0.535 | 0.339-0.73 | 0.636 | 0.5 |
| *Cardiobacterium valvarum* | 0.569 | 0.411-0.728 | 0.273 | 0.947 |
| *Granulicatella adiacens* | 0.571 | 0.355-0.786 | 0.364 | 0.921 |
| *Hoylesella* sp. HMT 317 | 0.572 | 0.411-0.732 | 0.273 | 0.947 |
| *Lancefieldella* sp. HMT 199 | 0.647 | 0.489-0.805 | 0.364 | 0.947 |
| *Porphyromonas catoniae* | 0.577 | 0.3951-0.7581 | 0.364 | 0.868 |
| *Lachnoanaerobaculum* sp. | 0.572 | 0.395-0.748 | 0.182 | 1.000 |
| *Eikenella corrodens* | 0.624 | 0.433-0.816 | 0.545 | 0.789 |
| *Staphylococcus warneri* | 0.589 | 0.438-0.739 | 0.182 | 1.000 |
| ***T5 enriched microbes*** | | | | |
| *Actinomyces naeslundii* | 0.487 | 0.19-0.784 | 0.571 | 0.727 |
| *Neisseria elongata* | 0.552 | 0.311-0.793 | 0.429 | 0.864 |
| *Cardiobacterium valvarum* | 0.688 | 0.470-0.907 | 0.429 | 1 |
| *Lachnoanaerobaculum saburreum* | 0.682 | 0.467-0.897 | 0.429 | 0.955 |
| *Leptotrichia* sp. HMT-417 | 0.61 | 0.408-0.812 | 0.286 | 1 |
| *Arachnia propionica* | 0.656 | 0.421-0.890 | 0.429 | 0.955 |
| *Leptotrichia hofstadii* | 0.61 | 0.408-0.812 | 0.286 | 1 |
| *Comamonas testosteroni* | 0.627 | 0.435-0.818 | 0.286 | 1 |
| *Lancefieldella parvula* | 0.656 | 0.446-0.865 | 0.571 | 0.818 |
| *Escherichia coli* | 0.610 | 0.383-0.837 | 0.429 | 0.818 |
| *Aggregatibacter* sp. HMT 898 | 0.627 | 0.435-0.818 | 0.286 | 1.000 |
| *Segatella oris* | 0.675 | 0.446-0.904 | 0.429 | 1.000 |
| *Prevotella veroralis* | 0.627 | 0.435-0.818 | 0.286 | 1.000 |
| *Hoylesella* sp. HMT 317 | 0.643 | 0.415-0.870 | 0.429 | 0.909 |

**Supplementary Table 6**: Persistence and resilience of microbial species from T1 and T2 samples to T5. The table lists species detected at T1 or T2 and retained at T5. count_S_P and count_S_NP represent persistent and non-persistent species in successful cases, while count_F_P and count_F_NP represent persistent and non-persistent species in failure cases. OR (Odds Ratio) indicates the likelihood of persistence, and p-value denotes statistical significance. “Inf” indicates species found exclusively in one group.

| **T1----> T5** | | | | | | |
| --- | --- | --- | --- | --- | --- | --- |
| **Species** | **count_S_P** | **count_S_NP** | **count_F_P** | **count_F_NP** | **OR** | **p_value** |
| *Fusobacterium polymorphum* | 8 | 10 | 7 | 0 | Inf | 0.020189 |
| *Rothia dentocariosa* | 16 | 2 | 3 | 4 | 0.09375 | 0.032411 |
| *Campylobacter showae* | 2 | 16 | 4 | 3 | 10.66667 | 0.032411 |
| *Corynebacterium durum* | 14 | 4 | 2 | 5 | 0.114286 | 0.058124 |
| *Lachnoanaerobaculum sp* | 3 | 15 | 4 | 3 | 6.666667 | 0.066362 |
| *Selenomonas felix* | 0 | 18 | 2 | 5 | Inf | 0.07 |
| *Aggregatibacter aphrophilus* | 0 | 18 | 2 | 5 | Inf | 0.07 |
| *Riemerella* sp. HMT-322 | 0 | 18 | 2 | 5 | Inf | 0.07 |
| *Cardiobacterium hominis* | 2 | 16 | 3 | 4 | 6 | 0.113043 |
| *Arachnia propionica* | 2 | 16 | 3 | 4 | 6 | 0.113043 |
| *Eikenella corrodens* | 2 | 16 | 3 | 4 | 6 | 0.113043 |
| *Gemella morbillorum* | 2 | 16 | 3 | 4 | 6 | 0.113043 |
| *Kluyvera ascorbata* | 6 | 12 | 0 | 7 | Inf | 0.137233 |
| *Schaalia* sp | 4 | 14 | 4 | 3 | 4.666667 | 0.15633 |
| *Leptotrichia buccalis* | 1 | 17 | 2 | 5 | 6.8 | 0.179565 |
| *Nanosynbacter fur* | 1 | 17 | 2 | 5 | 6.8 | 0.179565 |
| *Lachnoanaerobaculum saburreum* | 1 | 17 | 2 | 5 | 6.8 | 0.179565 |
| *Segatella oris* | 1 | 17 | 2 | 5 | 6.8 | 0.179565 |
| *Hoylesella loescheii* | 1 | 17 | 2 | 5 | 6.8 | 0.179565 |
| *Selenomonas artemidis* | 1 | 17 | 2 | 5 | 6.8 | 0.179565 |
| *Fusobacterium* sp | 7 | 11 | 5 | 2 | 3.928571 | 0.201556 |
| *Pseudomonas fluorescens* | 18 | 0 | 6 | 1 | 0 | 0.28 |
| *Staphylococcus epidermidis* | 0 | 18 | 1 | 6 | Inf | 0.28 |
| *Corynebacterium accolens* | 0 | 18 | 1 | 6 | Inf | 0.28 |
| *Peptidiphaga* sp. HMT-183 | 0 | 18 | 1 | 6 | Inf | 0.28 |
| *Scardovia wiggsiae* | 0 | 18 | 1 | 6 | Inf | 0.28 |
| *Cutibacterium namnetense* | 0 | 18 | 1 | 6 | Inf | 0.28 |
| *Lactobacillus* sp | 0 | 18 | 1 | 6 | Inf | 0.28 |
| *Pedobacter* sp. HMT-321 | 0 | 18 | 1 | 6 | Inf | 0.28 |
| *Aggregatibacter segnis* | 0 | 18 | 1 | 6 | Inf | 0.28 |
| *Lactobacillus iners* | 0 | 18 | 1 | 6 | Inf | 0.28 |
| *Lancefieldella sp* | 0 | 18 | 1 | 6 | Inf | 0.28 |
| *Prevotella pallens* | 0 | 18 | 1 | 6 | Inf | 0.28 |
| *Prevotella vespertina* | 0 | 18 | 1 | 6 | Inf | 0.28 |
| *Aggregatibacter* sp. HMT-513 | 0 | 18 | 1 | 6 | Inf | 0.28 |
| *Anaerovoracaceae [G1] sulci* | 0 | 18 | 1 | 6 | Inf | 0.28 |
| *Prevotella melaninogenica* | 0 | 18 | 1 | 6 | Inf | 0.28 |
| *Absconditicoccaceae [G1] bacterium* HMT-874 | 0 | 18 | 1 | 6 | Inf | 0.28 |
| *Capnocytophaga gingivalis* | 0 | 18 | 1 | 6 | Inf | 0.28 |
| *Gemella sanguinis* | 0 | 18 | 1 | 6 | Inf | 0.28 |
| *Selenomonas flueggei* | 0 | 18 | 1 | 6 | Inf | 0.28 |
| *Alloprevotella* sp. HMT-413 | 0 | 18 | 1 | 6 | Inf | 0.28 |
| *Capnocytophaga* sp. HMT-338 | 0 | 18 | 1 | 6 | Inf | 0.28 |
| *Moraxella* sp. HMT-276 | 0 | 18 | 1 | 6 | Inf | 0.28 |
| *Pseudomonas aeruginosa* | 0 | 18 | 1 | 6 | Inf | 0.28 |
| *Yersinia pestis* | 0 | 18 | 1 | 6 | Inf | 0.28 |
| *Hoylesella* sp. HMT-317 | 3 | 15 | 3 | 4 | 3.75 | 0.298498 |
| *Schaalia hongkongensis* | 3 | 15 | 3 | 4 | 3.75 | 0.298498 |
| *Actinomyces naeslundii* | 14 | 4 | 4 | 3 | 0.380952 | 0.355365 |
| *Porphyromonas pasteri* | 7 | 11 | 1 | 6 | 0.261905 | 0.362297 |
| *Veillonella parvula* | 6 | 12 | 4 | 3 | 2.666667 | 0.378126 |
| *Cutibacterium acnes* | 12 | 6 | 3 | 4 | 0.375 | 0.378126 |
| *Granulicatella adiacens* | 8 | 10 | 5 | 2 | 3.125 | 0.378261 |
| *Neisseria* sp | 17 | 1 | 6 | 1 | 0.352941 | 0.49 |
| *Streptococcus* sp. HMT-056 | 1 | 17 | 1 | 6 | 2.833333 | 0.49 |
| *Campylobacter concisus* | 1 | 17 | 1 | 6 | 2.833333 | 0.49 |
| *Acinetobacter johnsonii* | 1 | 17 | 1 | 6 | 2.833333 | 0.49 |
| *Dolosigranulum pigrum* | 1 | 17 | 1 | 6 | 2.833333 | 0.49 |
| *Actinomyces* sp. HMT-525 | 1 | 17 | 1 | 6 | 2.833333 | 0.49 |
| *Lancefieldella parvula* | 1 | 17 | 1 | 6 | 2.833333 | 0.49 |
| *Leptotrichia hofstadii* | 1 | 17 | 1 | 6 | 2.833333 | 0.49 |
| *Ruminococcaceae [G1] bacterium* HMT-075 | 1 | 17 | 1 | 6 | 2.833333 | 0.49 |
| *Segatella salivae* | 1 | 17 | 1 | 6 | 2.833333 | 0.49 |
| *Treponema vincentii* | 1 | 17 | 1 | 6 | 2.833333 | 0.49 |
| *Capnocytophaga* sp. HMT-878 | 1 | 17 | 1 | 6 | 2.833333 | 0.49 |
| *Porphyromonas endodontalis* | 1 | 17 | 1 | 6 | 2.833333 | 0.49 |
| *Treponema maltophilum* | 1 | 17 | 1 | 6 | 2.833333 | 0.49 |
| *Leptotrichia* sp. HMT-225 | 1 | 17 | 1 | 6 | 2.833333 | 0.49 |
| *Nanosyncoccus* sp. HMT-351 | 1 | 17 | 1 | 6 | 2.833333 | 0.49 |
| *Porphyromonas* sp | 1 | 17 | 1 | 6 | 2.833333 | 0.49 |
| *Weeksellaceae [G1] bacterium* HMT-907 | 1 | 17 | 1 | 6 | 2.833333 | 0.49 |
| *Escherichia coli* | 1 | 17 | 1 | 6 | 2.833333 | 0.49 |
| *Streptococcus sanguinis* | 15 | 3 | 7 | 0 | Inf | 0.534348 |
| *Actinomyces gerencseriae* | 3 | 15 | 0 | 7 | Inf | 0.534348 |
| *Streptococcus intermedius* | 3 | 15 | 0 | 7 | Inf | 0.534348 |
| *Enterobacter hormaechei* | 3 | 15 | 0 | 7 | Inf | 0.534348 |
| *Segatella oulorum* | 2 | 16 | 2 | 5 | 3.2 | 0.548458 |
| *Kingella oralis* | 2 | 16 | 2 | 5 | 3.2 | 0.548458 |
| *Lancefieldella* sp. HMT-199 | 2 | 16 | 2 | 5 | 3.2 | 0.548458 |
| *Capnocytophaga granulosa* | 2 | 16 | 2 | 5 | 3.2 | 0.548458 |
| *Cardiobacterium valvarum* | 2 | 16 | 2 | 5 | 3.2 | 0.548458 |
| *Leptotrichia* sp. HMT-417 | 2 | 16 | 2 | 5 | 3.2 | 0.548458 |
| *Pseudoleptotrichia* sp. HMT-215 | 2 | 16 | 2 | 5 | 3.2 | 0.548458 |
| *Selenomonas* sp | 2 | 16 | 2 | 5 | 3.2 | 0.548458 |
| *Peptostreptococcus stomatis* | 3 | 15 | 2 | 5 | 2 | 0.596838 |
| *Streptococcus gordonii* | 3 | 15 | 2 | 5 | 2 | 0.596838 |
| *Actinomyces dentalis* | 5 | 13 | 1 | 6 | 0.433333 | 0.637154 |
| *Paludibacteraceae [G1] bacterium* HMT-274 | 5 | 13 | 1 | 6 | 0.433333 | 0.637154 |
| *Veillonella* sp | 5 | 13 | 1 | 6 | 0.433333 | 0.637154 |
| *Actinomyces massiliensis* | 5 | 13 | 3 | 4 | 1.95 | 0.639559 |
| *Actinomyces* sp | 7 | 11 | 4 | 3 | 2.095238 | 0.656407 |
| *Corynebacterium matruchotii* | 8 | 10 | 2 | 5 | 0.5 | 0.659247 |
| *Lautropia mirabilis* | 8 | 10 | 4 | 3 | 1.666667 | 0.672769 |
| *Leptotrichia hongkongensis* | 6 | 12 | 3 | 4 | 1.5 | 0.672877 |
| *Abiotrophia defectiva* | 6 | 12 | 3 | 4 | 1.5 | 0.672877 |
| *Actinomyces oris* | 16 | 2 | 6 | 1 | 0.75 | 1 |
| *Neisseria elongata* | 5 | 13 | 2 | 5 | 1.04 | 1 |
| *Neisseria* sp. HMT-499 | 3 | 15 | 1 | 6 | 0.833333 | 1 |
| *Rothia aeria* | 13 | 5 | 5 | 2 | 0.961538 | 1 |
| *Streptococcus* sp | 17 | 1 | 7 | 0 | Inf | 1 |
| *Actinomyces* sp. HMT-448 | 1 | 17 | 0 | 7 | Inf | 1 |
| *Capnocytophaga leadbetteri* | 4 | 14 | 2 | 5 | 1.4 | 1 |
| *Capnocytophaga ochracea* | 6 | 12 | 2 | 5 | 0.8 | 1 |
| *Capnocytophaga sputigena* | 7 | 11 | 2 | 5 | 0.628571 | 1 |
| *Catonella morbi* | 2 | 16 | 0 | 7 | Inf | 1 |
| *Fusobacterium animalis* | 4 | 14 | 2 | 5 | 1.4 | 1 |
| *Leptotrichia wadei* | 4 | 14 | 2 | 5 | 1.4 | 1 |
| *Odoribacteraceae [G5] sp* | 1 | 17 | 0 | 7 | Inf | 1 |
| *Oribacterium* sp. HMT-078 | 2 | 16 | 1 | 6 | 1.333333 | 1 |
| *Parvisynbacter* sp. HMT-348 | 4 | 14 | 2 | 5 | 1.4 | 1 |
| *Prevotella micans* | 2 | 16 | 0 | 7 | Inf | 1 |
| *Pseudoleptotrichia* sp. HMT-212 | 4 | 14 | 2 | 5 | 1.4 | 1 |
| *Saccharimonas* sp. HMT-346 | 4 | 14 | 2 | 5 | 1.4 | 1 |
| *Schaalia georgiae* | 1 | 17 | 0 | 7 | Inf | 1 |
| *Segatella maculosa* | 1 | 17 | 0 | 7 | Inf | 1 |
| *Selenomonas noxia* | 4 | 14 | 2 | 5 | 1.4 | 1 |
| *Selenomonas sputigena* | 3 | 15 | 1 | 6 | 0.833333 | 1 |
| *Serratia marcescens* | 1 | 17 | 0 | 7 | Inf | 1 |
| *Staphylococcus sp* | 7 | 11 | 2 | 5 | 0.628571 | 1 |
| *Stomatobaculum longum* | 2 | 16 | 0 | 7 | Inf | 1 |
| *Tannerella serpentiformis* | 3 | 15 | 1 | 6 | 0.833333 | 1 |
| *Treponema* sp. HMT-268 | 1 | 17 | 0 | 7 | Inf | 1 |
| *Rothia mucilaginosa* | 1 | 17 | 0 | 7 | Inf | 1 |
| *Kocuria* sp | 1 | 17 | 0 | 7 | Inf | 1 |
| *Leptothrix* sp | 4 | 14 | 1 | 6 | 0.583333 | 1 |
| *Dialister invisus* | 2 | 16 | 1 | 6 | 1.333333 | 1 |
| *Olsenella* sp. HMT-807 | 2 | 16 | 1 | 6 | 1.333333 | 1 |
| *Prevotella denticola* | 2 | 16 | 1 | 6 | 1.333333 | 1 |
| *Prevotella nigrescens* | 3 | 15 | 1 | 6 | 0.833333 | 1 |
| *Fusobacterium vincentii* | 2 | 16 | 0 | 7 | Inf | 1 |
| *Haemophilus parainfluenzae* | 6 | 12 | 2 | 5 | 0.8 | 1 |
| *Leptothrix* sp. HMT-025 | 1 | 17 | 0 | 7 | Inf | 1 |
| *Selenomonas timonae* | 2 | 16 | 1 | 6 | 1.333333 | 1 |
| *Peptoanaerobacter yurii* | 3 | 15 | 1 | 6 | 0.833333 | 1 |
| *Streptococcus salivarius* | 2 | 16 | 1 | 6 | 1.333333 | 1 |
| *Aggregatibacter* sp. HMT-458 | 1 | 17 | 0 | 7 | Inf | 1 |
| *Aggregatibacter* sp. HMT-898 | 1 | 17 | 0 | 7 | Inf | 1 |
| *Alloprevotella tannerae* | 2 | 16 | 0 | 7 | Inf | 1 |
| *Anaerovoracaceae [G1] infirmum* | 1 | 17 | 0 | 7 | Inf | 1 |
| *Campylobacter gracilis* | 2 | 16 | 1 | 6 | 1.333333 | 1 |
| *Capnocytophaga bilenii* | 1 | 17 | 0 | 7 | Inf | 1 |
| *Capnocytophaga endodontalis* | 1 | 17 | 0 | 7 | Inf | 1 |
| *Enterobacter cancerogenus* | 7 | 11 | 2 | 5 | 0.628571 | 1 |
| *Fusobacterium hwasookii* | 1 | 17 | 0 | 7 | Inf | 1 |
| *Gallibacter brachy* | 1 | 17 | 0 | 7 | Inf | 1 |
| *Hoylesella marshii* | 1 | 17 | 0 | 7 | Inf | 1 |
| *Hoylesella saccharolytica* | 1 | 17 | 0 | 7 | Inf | 1 |
| *Johnsonella ignava* | 2 | 16 | 0 | 7 | Inf | 1 |
| *Kingella denitrificans* | 1 | 17 | 0 | 7 | Inf | 1 |
| *Lachnospiraceae [G3] bacterium* HMT-100 | 2 | 16 | 0 | 7 | Inf | 1 |
| *Lacticaseibacillus* sp | 2 | 16 | 0 | 7 | Inf | 1 |
| *Leptotrichia shahii* | 4 | 14 | 1 | 6 | 0.583333 | 1 |
| *Nanosynbacter* sp | 2 | 16 | 0 | 7 | Inf | 1 |
| *Neisseria* sp. HMT-020 | 1 | 17 | 0 | 7 | Inf | 1 |
| *Parvimonas micra* | 1 | 17 | 0 | 7 | Inf | 1 |
| *Parvimonas* sp | 1 | 17 | 0 | 7 | Inf | 1 |
| *Peptidiphaga gingivicola* | 1 | 17 | 0 | 7 | Inf | 1 |
| *Peptococcus* sp. HMT-167 | 1 | 17 | 0 | 7 | Inf | 1 |
| *Peptococcus* sp. HMT-168 | 1 | 17 | 0 | 7 | Inf | 1 |
| *Porphyromonas* sp. HMT-275 | 2 | 16 | 0 | 7 | Inf | 1 |
| *Porphyromonas* sp. HMT-930 | 1 | 17 | 0 | 7 | Inf | 1 |
| *Pseudoleptotrichia* sp | 1 | 17 | 0 | 7 | Inf | 1 |
| *Pseudoleptotrichia* sp. HMT-219 | 1 | 17 | 0 | 7 | Inf | 1 |
| *Saccharimonas* sp. HMT-347 | 2 | 16 | 0 | 7 | Inf | 1 |
| *Schaalia* sp. HMT-180 | 3 | 15 | 1 | 6 | 0.833333 | 1 |
| *Solobacterium moorei* | 1 | 17 | 0 | 7 | Inf | 1 |
| *Streptococcus parasanguinis* | 1 | 17 | 0 | 7 | Inf | 1 |
| *Treponema socranskii* | 1 | 17 | 0 | 7 | Inf | 1 |
| *Treponema* sp. HMT-237 | 1 | 17 | 0 | 7 | Inf | 1 |
| *Capnocytophaga* sp. HMT-901 | 2 | 16 | 0 | 7 | Inf | 1 |
| *Micrococcus luteus* | 4 | 14 | 1 | 6 | 0.583333 | 1 |
| *Neisseria mucosa* | 1 | 17 | 0 | 7 | Inf | 1 |
| *Cupriavidus gilardii* | 1 | 17 | 0 | 7 | Inf | 1 |
| *Pedobacter sp* | 1 | 17 | 0 | 7 | Inf | 1 |
| *Actinomyces israelii* | 1 | 17 | 0 | 7 | Inf | 1 |
| *Arthrospira platensis* | 1 | 17 | 0 | 7 | Inf | 1 |
| *Hoylesella oralis* | 1 | 17 | 0 | 7 | Inf | 1 |
| *Leptotrichia* sp. HMT-498 | 1 | 17 | 0 | 7 | Inf | 1 |
| *Mitsuokella* sp. HMT-521 | 1 | 17 | 0 | 7 | Inf | 1 |
| *Nanogingivalis* sp. HMT-399 | 1 | 17 | 0 | 7 | Inf | 1 |
| *Odoribacteraceae [G5] bacterium* HMT-511 | 1 | 17 | 0 | 7 | Inf | 1 |
| *Prevotella* sp | 1 | 17 | 0 | 7 | Inf | 1 |
| *Prevotella veroralis* | 1 | 17 | 0 | 7 | Inf | 1 |
| *Pseudoleptotrichia* sp. HMT-392 | 1 | 17 | 0 | 7 | Inf | 1 |
| *Saccharimonas* sp. HMT-349 | 1 | 17 | 0 | 7 | Inf | 1 |
| *Streptococcus anginosus* | 2 | 16 | 0 | 7 | Inf | 1 |
| *Streptococcus mitis* | 1 | 17 | 0 | 7 | Inf | 1 |
| *Tannerella forsythia* | 2 | 16 | 0 | 7 | Inf | 1 |
| *Oribacterium sinus* | 1 | 17 | 0 | 7 | Inf | 1 |
| *Acidovorax temperans* | 2 | 16 | 0 | 7 | Inf | 1 |
| *Corynebacterium* sp | 2 | 16 | 0 | 7 | Inf | 1 |
| *Streptococcus mutans* | 1 | 17 | 0 | 7 | Inf | 1 |
| *Delftia acidovorans* | 1 | 17 | 0 | 7 | Inf | 1 |
| *Actinomyces* sp. HMT-896 | 1 | 17 | 0 | 7 | Inf | 1 |
| *Afipia broomeae* | 1 | 17 | 0 | 7 | Inf | 1 |
| *Saccharimonadales [F4 G1] bacterium* HMT-954 | 1 | 17 | 0 | 7 | Inf | 1 |

| **T2----> T5** | | | | | | |
| --- | --- | --- | --- | --- | --- | --- |
| **Species** | **count_S_P** | **count_S_NP** | **count_F_P** | **count_F_NP** | **OR** | **p_value** |
| *Rothia dentocariosa* | 14 | 4 | 1 | 6 | 0.047619 | 0.006803 |
| *Selenomonas* sp | 0 | 18 | 3 | 4 | Inf | 0.015217 |
| *Corynebacterium durum* | 13 | 5 | 1 | 6 | 0.064103 | 0.021281 |
| *Campylobacter showae* | 2 | 16 | 4 | 3 | 10.66667 | 0.032411 |
| *Peptostreptococcus stomatis* | 1 | 17 | 3 | 4 | 12.75 | 0.052569 |
| *Lancefieldella* sp. HMT-199 | 0 | 18 | 2 | 5 | Inf | 0.07 |
| *Oribacterium sp. HMT-078* | 0 | 18 | 2 | 5 | Inf | 0.07 |
| *Corynebacterium matruchotii* | 6 | 12 | 0 | 7 | Inf | 0.137233 |
| *Porphyromonas pasteri* | 6 | 12 | 0 | 7 | Inf | 0.137233 |
| *Pseudomonas fluorescens* | 17 | 1 | 5 | 2 | 0.147059 | 0.179565 |
| *Treponema socranskii* | 1 | 17 | 2 | 5 | 6.8 | 0.179565 |
| *Segatella oris* | 1 | 17 | 2 | 5 | 6.8 | 0.179565 |
| *Veillonella parvula* | 5 | 13 | 4 | 3 | 3.466667 | 0.20491 |
| *Streptococcus sanguinis* | 13 | 5 | 7 | 0 | Inf | 0.274308 |
| *Anaerovoracaceae [G4] bacterium* HMT-103 | 0 | 18 | 1 | 6 | Inf | 0.28 |
| *Gallibacter brachy* | 0 | 18 | 1 | 6 | Inf | 0.28 |
| *Odoribacteraceae [G3] bacterium* HMT-365 | 0 | 18 | 1 | 6 | Inf | 0.28 |
| *Odoribacteraceae [G5] bacterium* HMT-511 | 0 | 18 | 1 | 6 | Inf | 0.28 |
| *Peptidiphaga* sp. HMT-183 | 0 | 18 | 1 | 6 | Inf | 0.28 |
| *Peptococcus* sp | 0 | 18 | 1 | 6 | Inf | 0.28 |
| *Prevotella micans* | 0 | 18 | 1 | 6 | Inf | 0.28 |
| *Selenomonadaceae [G1] bacterium* HMT-135 | 0 | 18 | 1 | 6 | Inf | 0.28 |
| *Treponema denticola* | 0 | 18 | 1 | 6 | Inf | 0.28 |
| *Treponema lecithinolyticum* | 0 | 18 | 1 | 6 | Inf | 0.28 |
| *Treponema vincentii* | 0 | 18 | 1 | 6 | Inf | 0.28 |
| *Capnocytophaga* sp. HMT-878 | 0 | 18 | 1 | 6 | Inf | 0.28 |
| *Treponema maltophilum* | 0 | 18 | 1 | 6 | Inf | 0.28 |
| *Absconditicoccaceae [G1] bacterium* HMT-874 | 0 | 18 | 1 | 6 | Inf | 0.28 |
| *Aggregatibacter aphrophilus* | 0 | 18 | 1 | 6 | Inf | 0.28 |
| *Capnocytophaga granulosa* | 0 | 18 | 1 | 6 | Inf | 0.28 |
| *Fusobacterium hwasookii* | 0 | 18 | 1 | 6 | Inf | 0.28 |
| *Riemerella* sp. HMT-322 | 0 | 18 | 1 | 6 | Inf | 0.28 |
| *Selenomonas artemidis* | 0 | 18 | 1 | 6 | Inf | 0.28 |
| *Anaerovoracaceae [G1] sulci* | 0 | 18 | 1 | 6 | Inf | 0.28 |
| *Catonella morbi* | 0 | 18 | 1 | 6 | Inf | 0.28 |
| *Lachnospiraceae [G8] bacterium* HMT-500 | 0 | 18 | 1 | 6 | Inf | 0.28 |
| *Selenomonas timonae* | 0 | 18 | 1 | 6 | Inf | 0.28 |
| *Leptotrichia hongkongensis* | 4 | 14 | 0 | 7 | Inf | 0.294466 |
| *Streptococcus* sp | 15 | 3 | 4 | 3 | 0.266667 | 0.298498 |
| *Fusobacterium animalis* | 3 | 15 | 3 | 4 | 3.75 | 0.298498 |
| *Staphylococcus* sp | 7 | 11 | 1 | 6 | 0.261905 | 0.362297 |
| *Fusobacterium polymorphum* | 8 | 10 | 5 | 2 | 3.125 | 0.378261 |
| *Rothia aeria* | 10 | 8 | 2 | 5 | 0.32 | 0.378261 |
| *Actinomyces naeslundii* | 10 | 8 | 2 | 5 | 0.32 | 0.378261 |
| *Selenomonas noxia* | 1 | 17 | 1 | 6 | 2.833333 | 0.49 |
| *Dolosigranulum pigrum* | 1 | 17 | 1 | 6 | 2.833333 | 0.49 |
| *Porphyromonas endodontalis* | 1 | 17 | 1 | 6 | 2.833333 | 0.49 |
| *Streptococcus salivarius* | 1 | 17 | 1 | 6 | 2.833333 | 0.49 |
| *Gemella morbillorum* | 1 | 17 | 1 | 6 | 2.833333 | 0.49 |
| *Johnsonella ignava* | 1 | 17 | 1 | 6 | 2.833333 | 0.49 |
| *Lachnoanaerobaculum saburreum* | 1 | 17 | 1 | 6 | 2.833333 | 0.49 |
| *Schaalia hongkongensis* | 1 | 17 | 1 | 6 | 2.833333 | 0.49 |
| *Dialister invisus* | 1 | 17 | 1 | 6 | 2.833333 | 0.49 |
| *Oribacterium sinus* | 1 | 17 | 1 | 6 | 2.833333 | 0.49 |
| *Leptotrichia wadei* | 3 | 15 | 0 | 7 | Inf | 0.534348 |
| *Neisseria elongata* | 3 | 15 | 0 | 7 | Inf | 0.534348 |
| *Yersinia pestis* | 3 | 15 | 0 | 7 | Inf | 0.534348 |
| *Pseudoleptotrichia* sp. HMT-212 | 2 | 16 | 2 | 5 | 3.2 | 0.548458 |
| *Lachnoanaerobaculum* sp | 2 | 16 | 2 | 5 | 3.2 | 0.548458 |
| *Eikenella corrodens* | 3 | 15 | 2 | 5 | 2 | 0.596838 |
| *Peptoanaerobacter yurii* | 3 | 15 | 2 | 5 | 2 | 0.596838 |
| *Lautropia mirabilis* | 6 | 12 | 1 | 6 | 0.333333 | 0.625696 |
| *Enterobacter cancerogenus* | 6 | 12 | 1 | 6 | 0.333333 | 0.625696 |
| *Abiotrophia defectiva* | 5 | 13 | 1 | 6 | 0.433333 | 0.637154 |
| *Schaalia* sp | 5 | 13 | 1 | 6 | 0.433333 | 0.637154 |
| *Fusobacterium* sp | 5 | 13 | 3 | 4 | 1.95 | 0.639559 |
| *Cutibacterium acnes* | 8 | 10 | 2 | 5 | 0.5 | 0.659247 |
| *Actinomyces oris* | 14 | 4 | 5 | 2 | 0.714286 | 1 |
| *Neisseria* sp | 15 | 3 | 6 | 1 | 1.2 | 1 |
| *Campylobacter concisus* | 1 | 17 | 0 | 7 | Inf | 1 |
| *Capnocytophaga ochracea* | 4 | 14 | 2 | 5 | 1.4 | 1 |
| *Saccharimonas* sp. HMT-346 | 2 | 16 | 0 | 7 | Inf | 1 |
| *Tannerella serpentiformis* | 1 | 17 | 0 | 7 | Inf | 1 |
| *Fusobacterium vincentii* | 2 | 16 | 1 | 6 | 1.333333 | 1 |
| *Paludibacteraceae [G1] bacterium* HMT-274 | 6 | 12 | 2 | 5 | 0.8 | 1 |
| *Tannerella forsythia* | 2 | 16 | 1 | 6 | 1.333333 | 1 |
| *Corynebacterium* sp | 3 | 15 | 1 | 6 | 0.833333 | 1 |
| *Granulicatella adiacens* | 7 | 11 | 3 | 4 | 1.178571 | 1 |
| *Neisseriaceae [G1] bacterium* HMT-174 | 1 | 17 | 0 | 7 | Inf | 1 |
| *Rothia mucilaginosa* | 1 | 17 | 0 | 7 | Inf | 1 |
| *Acinetobacter johnsonii* | 2 | 16 | 0 | 7 | Inf | 1 |
| *Actinomyces* sp | 4 | 14 | 1 | 6 | 0.583333 | 1 |
| *Staphylococcus aureus* | 1 | 17 | 0 | 7 | Inf | 1 |
| *Actinomyces dentalis* | 2 | 16 | 0 | 7 | Inf | 1 |
| *Actinomyces massiliensis* | 3 | 15 | 1 | 6 | 0.833333 | 1 |
| *Actinomyces* sp. HMT-525 | 1 | 17 | 0 | 7 | Inf | 1 |
| *Aggregatibacter* sp. HMT-898 | 1 | 17 | 0 | 7 | Inf | 1 |
| *Alloprevotella tannerae* | 1 | 17 | 0 | 7 | Inf | 1 |
| *Anaerovoracaceae [G1] infirmum* | 1 | 17 | 0 | 7 | Inf | 1 |
| *Arachnia propionica* | 2 | 16 | 1 | 6 | 1.333333 | 1 |
| *Campylobacter gracilis* | 2 | 16 | 1 | 6 | 1.333333 | 1 |
| *Capnocytophaga endodontalis* | 1 | 17 | 0 | 7 | Inf | 1 |
| *Capnocytophaga sputigena* | 3 | 15 | 1 | 6 | 0.833333 | 1 |
| *Cardiobacterium hominis* | 3 | 15 | 1 | 6 | 0.833333 | 1 |
| *Cardiobacterium valvarum* | 2 | 16 | 1 | 6 | 1.333333 | 1 |
| *Haemophilus parainfluenzae* | 4 | 14 | 2 | 5 | 1.4 | 1 |
| *Hoylesella loescheii* | 1 | 17 | 0 | 7 | Inf | 1 |
| *Hoylesella* sp. HMT-317 | 1 | 17 | 0 | 7 | Inf | 1 |
| *Kingella denitrificans* | 1 | 17 | 0 | 7 | Inf | 1 |
| *Lachnospiraceae [G3] bacterium* HMT-100 | 2 | 16 | 0 | 7 | Inf | 1 |
| *Lactobacillus* sp | 2 | 16 | 1 | 6 | 1.333333 | 1 |
| *Lactococcus lactis* | 1 | 17 | 0 | 7 | Inf | 1 |
| *Leptothrix* sp | 2 | 16 | 0 | 7 | Inf | 1 |
| *Leptotrichia shahii* | 3 | 15 | 1 | 6 | 0.833333 | 1 |
| *Leptotrichia* sp. HMT-225 | 1 | 17 | 0 | 7 | Inf | 1 |
| *Nanosynbacter fur* | 1 | 17 | 0 | 7 | Inf | 1 |
| *Nanosynbacter* sp | 1 | 17 | 0 | 7 | Inf | 1 |
| *Neisseria* sp. HMT-020 | 1 | 17 | 0 | 7 | Inf | 1 |
| *Olsenella* sp. HMT-807 | 1 | 17 | 0 | 7 | Inf | 1 |
| *Pedobacter* sp. HMT-321 | 1 | 17 | 0 | 7 | Inf | 1 |
| *Peptidiphaga gingivicola* | 1 | 17 | 0 | 7 | Inf | 1 |
| *Peptococcus* sp. HMT-167 | 1 | 17 | 0 | 7 | Inf | 1 |
| *Porphyromonas* sp. HMT-275 | 1 | 17 | 0 | 7 | Inf | 1 |
| *Prevotella denticola* | 2 | 16 | 0 | 7 | Inf | 1 |
| *Prevotella nigrescens* | 2 | 16 | 1 | 6 | 1.333333 | 1 |
| *Pseudoleptotrichia* sp | 2 | 16 | 0 | 7 | Inf | 1 |
| *Pseudoleptotrichia* sp. HMT-219 | 1 | 17 | 0 | 7 | Inf | 1 |
| *Saccharimonas* sp. HMT-347 | 1 | 17 | 0 | 7 | Inf | 1 |
| *Schaalia* sp. HMT-180 | 3 | 15 | 1 | 6 | 0.833333 | 1 |
| *Selenomonas sputigena* | 3 | 15 | 1 | 6 | 0.833333 | 1 |
| *Streptococcus gordonii* | 2 | 16 | 0 | 7 | Inf | 1 |
| *Streptococcus intermedius* | 1 | 17 | 0 | 7 | Inf | 1 |
| *Veillonella* sp | 4 | 14 | 2 | 5 | 1.4 | 1 |
| *Arthrospira platensis* | 1 | 17 | 0 | 7 | Inf | 1 |
| *Capnocytophaga* sp. HMT-901 | 1 | 17 | 0 | 7 | Inf | 1 |
| *Enterobacter hormaechei* | 2 | 16 | 1 | 6 | 1.333333 | 1 |
| *Enterococcus faecalis* | 1 | 17 | 0 | 7 | Inf | 1 |
| *Kingella oralis* | 4 | 14 | 1 | 6 | 0.583333 | 1 |
| *Kluyvera ascorbata* | 3 | 15 | 1 | 6 | 0.833333 | 1 |
| *Kocuria* sp | 2 | 16 | 0 | 7 | Inf | 1 |
| *Microbacterium ginsengisoli* | 1 | 17 | 0 | 7 | Inf | 1 |
| *Pedobacter* sp | 1 | 17 | 0 | 7 | Inf | 1 |
| *Solobacterium moorei* | 1 | 17 | 0 | 7 | Inf | 1 |
| *Actinomyces israelii* | 1 | 17 | 0 | 7 | Inf | 1 |
| *Aggregatibacter* sp | 1 | 17 | 0 | 7 | Inf | 1 |
| *Capnocytophaga leadbetteri* | 1 | 17 | 0 | 7 | Inf | 1 |
| *Hoylesella oralis* | 1 | 17 | 0 | 7 | Inf | 1 |
| *Leptotrichia buccalis* | 1 | 17 | 0 | 7 | Inf | 1 |
| *Leptotrichia hofstadii* | 1 | 17 | 0 | 7 | Inf | 1 |
| *Leptotrichia* sp. HMT-498 | 1 | 17 | 0 | 7 | Inf | 1 |
| *Pseudoleptotrichia* sp. HMT-215 | 1 | 17 | 0 | 7 | Inf | 1 |
| *Pseudoleptotrichia* sp. HMT-392 | 1 | 17 | 0 | 7 | Inf | 1 |
| *Streptococcus anginosus* | 2 | 16 | 0 | 7 | Inf | 1 |
| *Staphylococcus warneri* | 1 | 17 | 0 | 7 | Inf | 1 |
| *Parvisynbacter* sp. HMT-348 | 2 | 16 | 0 | 7 | Inf | 1 |
| *Porphyromonas catoniae* | 3 | 15 | 1 | 6 | 0.833333 | 1 |
| *Streptococcus mutans* | 2 | 16 | 0 | 7 | Inf | 1 |
| *Clostridiales [F3 G1] bacterium* HMT-876 | 1 | 17 | 0 | 7 | Inf | 1 |
| *Staphylococcus hominis* | 1 | 17 | 0 | 7 | Inf | 1 |
| *Serratia marcescens* | 1 | 17 | 0 | 7 | Inf | 1 |
