# Supplementary material for: Distinct Microbiota and Functional Pathway Profiles Define Success and Failure in Regenerative Endodontic Treatment
Source: Int Endod J. 2026 Jun 23;59(10):2223–37. doi: 10.1111/iej.70205 (PMC13569218; doi:10.1111/iej.70205)
Supplement: Supplementary file 2 [file IEJ-59-2223-s003.docx]

**Supplementary Figure**

**Fig. S1**. Taxonomic composition of microbial communities across time points and treatment outcomes.

**a** Sample-wise stacked bar plot depicting the relative abundance of the top phyla across all samples from T1-T5. Each bar represents an individual sample ordered by time point; low-abundance taxa are grouped as “Other.” **b** Sample-wise stacked bar plots showing the relative abundance of the top families across all samples from T1-T5. Each bar represents an individual sample ordered by time point; remaining taxa are aggregated as “Other.” **c** Sample-wise stacked bar plots at the phylum level stratified by treatment outcome (Success vs Failure). Samples are organized across time points (T1–T5), with each bar representing an individual sample. Low-abundance taxa are grouped as “Other.”
